# Normative values to Horus® computerized posturography in children

**DOI:** 10.1590/2317-1782/20242023241en

**Published:** 2024-08-05

**Authors:** Ândrea de Melo Boaz, Rudimar dos Santos Riesgo, Pricila Sleifer

**Affiliations:** 1 Universidade Federal do Rio Grande do Sul – UFRGS - Porto Alegre (RS), Brasil.

**Keywords:** Postural Balance, Child, Proprioception, Reference Standards, Validation Study, Growth and Development, Vestibular System, Diagnostic Equipment

## Abstract

**Purpose:**

Propose normalization values of the Horus® computerized posturography platform, in children aged 4 to 6 years, without auditory and/or vestibular complaints.

**Methods:**

Cross-sectional study, 216 children aged 4 to 6 years participated. All the children underwent to visual screening, audiological evaluation and computerized posturography, which consists of research on stability limits and seven sensory conditions. The results were statistically analyzed using the tests non-parametric Kruskal-Walli, post hoc Dunn-Bonferroni for pairwise age comparisons and the Mann-Whitney U for sex analysis. Categorical data were presented in relative frequency and quantitative data in mean and standard deviation.

**Results:**

Standardization values were described for the stability limit and for the seven sensory conditions. There was a difference for the stability limit between sex at 4 years old(p<0.007) and, in the comparison between ages 4 and 5 (p=0.005) and 4 and 6 years old(p<0.001). In the residual functional balance, comparison between ages, there was a difference between 4 and 5, 4 and 6, 5 and 6 years, however for different data. The presence of statistical difference for different evaluation data also occurred in the analysis by sex. In the sensory systems, the findings between ages showed differences for the vestibular system, right and left optokinetic visual dependence, tunnel visual dependence and for the composite balance index.

**Conclusion:**

It was possible to establish normative values for the Horus® posturography in healthy children aged 4 to 6 years.

## INTRODUCTION

The assessment of body balance can be performed using a functional approach, according to the most appropriate selection at the time of the assessment, chosen by the examiner, and can be performed across systems by analyzing biomechanics, cognitively motor and sensory organization or, through posturographs^(1)^, in a qualitative observational or quantitative way, using measuring instruments^(2)^.

Generally, posturography is used to complement the assessment of body balance, along with other procedures such as the vectoelectronystagmography exam^(3)^. This happens because these exams have different objectives, and posturography allows the evaluation of body sway under different test conditions, generating responses to the visual, vestibular and somatosensory systems, important in maintaining body balance^(3,4)^. In this way, the answers obtained with the posturography exam allow the perception of how the functional ability is, being a useful instrument for understanding the appropriate use of information from the vestibular system by the subject^(4)^.

In children, the assessment of the vestibular system becomes more challenging, due to the presence of difficulty in reporting by the child and thus, in identifying complaints^(5)^. Often, dizziness may not be understood as a symptom and could be described as a report of pain, a hysterical episode, or simply behavior interpreted as stubbornness and annoyance^(5)^. Furthermore, there are limitations in the assessment procedures in this population^(6)^. In this context, computerized posturography with the aim to facilitate and expedite the evaluation of body control in children under various in different conditions^(7)^.

For the evaluation with computerized posturography, a force platform is used which allows verification of the oscillations of the gravity center and the pressure center (PC), generated by the subject positioned under the platform^(8)^. In the posturographic analysis, many variables can be derived from the statokinesigram and stabilogram, in the center of pressure, generated during the exam^(2)^. These analyses can be divided into two types: global analysis, which measures the oscillation patterns both in the time domain and in the frequency domain; and structural analysis, which identifies subunits in posturographic data and relates them to motor control processes^(2)^.

The Horus® computerized posturography platform presents high precision to determine the center of body pressure^(9)^, which is the point of application of the resultant of the vertical forces acting on the support surface, and its result depends on the task that is investigated during the evaluation^(2)^. Thus, when the subject remains standing and static on the force platform during the test, the presence of small oscillations of the pressure center are observed^(2)^.

Recently, researchers have proposed normative values for the Horus® computerized platform in adults, covering ages 40 to 59 years^(10)^ and 20 to 89 years^(11)^, revealing age and sex differences for various variables. However, there are no publications yet on normative values for children using this equipment^(7)^. Generally, there are rare studies on the use of posturography in children^(12-14)^, statement also highlighted previously by the North American Academy of Neurology^(15)^. Therefore, there is a scarcity of publications suggesting normative values for static posturography, especially in young children, despite it being a tool frequently used to assess body balance.

Given the above, the purpose of this study was to describe a proposal for standardization of the Horus® computerized posturography platform, in children aged 4 to 6 years, without complaints related to hearing and body balance, with typical development, through quantitative and descriptive statistical analysis, in the verification by age and sex.

## METHODS

This is a cross-sectional study with a quantitative approach, approved by the Ethics Committee of a Public Education Institution, under number 39835. In addition, this study complied with the regulatory norms and guidelines for research with human beings of Resolution 466/12 of the National Health Council. For the consent of those responsible for the children to participate in the research, the Term of Free and Informed Consent was delivered, requiring the signature of the same by those responsible, and the consent of the participant through the Term of Assent of the Child for those who were already able to to sign, as well as, consent to the use of images was requested.

The sample was made up of children aged between 4 years and 6 years and 11 months, of both sexes, from daycare centers and municipal and private public schools in the city of Santiago-RS. The children did not have complaints related to the vestibular and/or auditory system and had typical development. Recruitment of participants involved various methods of outreach, including local newspapers, social media platforms, blogs, radio announcements, and direct communication with schools.

The sample size was determined using a non-probabilistic convenience sampling method, based on a standardized effect size of 0.9. The estimated calculation resulted in a sample size of 192 children. The study adopted a significance level of 0.05 with a statistical power of 95% (Epi Info – StatCalc).

The inclusion criteria for the subjects of the research were: age between 4 and 6 years and 11 months, presenting typical development, without otoneurological and/or auditory complaints. Typical development was considered to be a child who presented progress as expected for the age group, considering the developmental milestones in the gross motor, fine motor, language and speech, cognitive and social/emotional growth domains, reported in the anamnesis with parents/guardians.

The following exclusion criteria were listed: Children with syndromes or craniofacial abnormalities; body weight lower than 15 kg; presenting otological alterations, headaches; having a history of frequent falls or presence of dizziness, vertigo, kinetosis; having visual difficulties and/or motor impairment, which could compromise the performance of the assessment; making use of medicine with action on the vestibular system or central nervous system; not understanding or being unable, for whatever reason, to carry out the procedures and complete the assessment battery.

The first stage of evaluation was taking an anamnesis with their parents/guardians to understand the child's development up to the moment of the assessment, as well as checking for possible hearing complaints and/or body balance.

Afterwards, anthropometric measurements were verified, such as the child's height, using a measuring tape fixed to the wall of the evaluation room, and weight, measured on a digital scale, which was later confirmed during the balance test using the Horus® platform. For visual acuity screening, Snellen's “E” directional optotype was used, following the guidelines for using the tool according to the Ministry of Health^(16)^.

The peripheral auditory assessment was performed by means of visual inspection of the external auditory meatus and by pure tone audiometry tests, using the screening method (scanning method^(17)^ at an 20dB intensity at frequencies of 1000, 2000 and 4000 Hz with addition of bass frequency 500 Hz, in both ears, assessed individually (carried out with Callisto equipment, in an acoustic booth, using a TDH-39 earphone), children who responded to all frequencies are considered eligible; Screening transient otoacoustic emissions (AccuScreen equipment through insert earphones), there must be a response in both ears; 226Hz probe tympanometry with investigation of contralateral acoustic reflexes at frequencies of 500, 1000, 2000 and 4000 Hz (Zodiac equipment with insert earphones and probe), with adequate responses being the presence of a type A tympanometric curve. All equipment was calibrated according to ISO 8253-1 standards.

The last exam performed was the computerized posturography, with Horus® equipment, from the company Contronic, adapted especially for this research by the manufacturer due to the need to adjust the minimum weight for the study population. The Horus® posturography has a “Strength Platform” which allows the determination of the Center of Pressure (CP) by means of four force sensors arranged in a rectangular position. These sensors enable the measurement of force components in the anteroposterior, mediolateral and vertical directions, acting on the platform. This is connected via USB to a notebook containing the previously installed Horus® software, which records the parameters in the time and frequency domain and displays the responses on the computer, generating quantitative and objective data through posturography tests. In this study, an Asus X450CA notebook was used.

The images needed to carry out the evaluation were presented on a 40-inch Sony television, set at a distance of 1 meter from the platform and the child's positioning point, located so that the screen was at eye level. To carry out the evaluation, the child was instructed to take off his/her shoes, remaining barefoot; climb on a 5 cm high platform; remain erect with feet apart and comfortable for 30 seconds, aligned on the horizontal line of the platform, symmetrically distanced from the anteroposterior line; the hallux finger pointing between 0 and 15 degrees; keeping eyes open looking at the television; look at the images that will appear and finally, keeping the eyes closed. Afterwards, a cushion measuring 5cm in height and density D33 was added, staying 10cm from the floor, with eyes open and then with eyes closed, being the child guided again in the new examination condition. The complete evaluation consisted of eight stages, as follows:

1. Limit of Stability Test, (SL) in platform without cushion, the child had to climb on the platform and lean the body forward, returning to the center, leaning backwards, returning to the center, leaning the body to the right, returning to the center, leaning the body to the left, returning to the center and repeat. The movement only of the ankles should take place without hurry, without moving the hips or shoulders and without removing the feet from the platform. In this step, the extreme points in each direction recorded by the movement of the body, allowed to define the value of the stability ellipse, which served as a reference for the other sequential steps;
  1. Sensory condition 1, platform without cushion: standing with eyes open, static, looking at a fixed yellow dot of 10% size against a black background, projected on television;
  2. Sensory condition 2, platform without cushion: standing with eyes closed, not moving;
  3. Sensory Condition 3, Platform with added cushion: standing with eyes open, static, looking at a fixed yellow dot of 10% size against a black background, projected on television;
  4. Sensory condition 4, platform with added cushion: standing with eyes closed, not moving;
  5. Sensory condition 5, platform with added cushion: remain standing, static, watching on television the video of bars in black and white colors in horizontal optokinetic effect moving to the right, at a speed of 10%;
  6. Sensory condition 6, platform with added cushion: remain standing, static, watching a video on television containing bars in black and white in horizontal optokinetic effect moving to the left, at a speed of 10%;
  7. Sensory condition 7, platform with added cushion: remain standing, static, watching a video on television of a tunnel made up of thin bars moving forward at a speed of 4%, without rotation.

**Figure 1:**
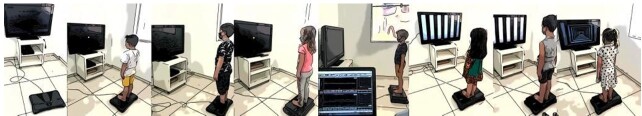
Image of the positioning of the Horus® platform in the evaluation room and in the sequence of exam conditions 1, 2, 3, 4, 5, 6, and 7, respectively

The guidelines for the above assessment steps were provided in simple language, aiming for easy compression of the children. Furthermore, the examiner demonstrated to the child the movements required to record the SL and the “still” position in the other test conditions before conducting the assessment. In Figure 1, it is possible to observe the positioning of the evaluation room equipment and the position of the child in the seven test conditions:

Strategies were used to keep the children immobile, such as the aid of a doll positioned on the floor next to the platform, “in a statue position”, simulating the assessment process alongside the child; given to the child the suggestion that when looking at the image presented on the television screen, without moving, a “chick” could appear behind the yellow ball in conditions 1 and 3, and a “zebra” in conditions 5, 6 and 7; family members/companions were also encouraged to participate by “playing statues” and conducting assessments close to the child. Additionally, normal countdown or countdown aloud, to mark the time of 30 seconds.

The evaluation was carried out in the presence of two evaluators, who monitored the child's behavior during the evaluation and analyzed the responses separately, aiming to improve the reliability of the findings. Subsequently, the quantified data were tabulated. Upon identifying any alterations in the assessments, the parents/guardians were guided and the child referred for the appropriate treatments and complementary evaluations.

The values of postural oscillations under the platform were registered during the time of performance in each test condition by the stabilogram, in the direction of mediolateral movement to the left and to the right, and anteroposterior forward and backward, by the amplitude measure. For the statokinesigram, there is a map referring to the displacement of the PC in the mediolateral axis in relation to the displacement of the PC in the anteroposterior axis, with a cloud of 95% confidence ellipse points.

In this study, the data analyzed in the responses obtained on the platform were: LS, which corresponds to the extent to which the body can move in relation to the center of gravity without having to change its support base, and the LS area which refers to the region where the body can perform such an action, in mm^2^; Confidence ellipse (CE), characterized as the quantitative record of displacement/difficulty of the subject in keeping himself without oscillation in relation to the PC, in mm^2^; Speed, that refers to the average speed in relation to the PC, the lower its value, in mm/s, the better the body balance; and the Residual Functional Balance (RFB), which demonstrates the area still available, safely, for the subject's oscillation recorded in percentage, by the ratio between the SL area and the Confidence Ellipse area.

Data were analyzed using the Statistical Package for Social Sciences (SPSS) software for Windows, version 22.0. Categorical data were presented in relative frequency, and quantitative data by mean and standard deviation. The Kruskal-Walli non-parametric statistical test was used, the post hoc Dunn-Bonferroni test for pairwise age comparisons and the Mann-Whitney U test for analysis between sex. Values of p<0.05 were considered significant.

## RESULTS

A total of 231 children attended the evaluation, 15 of whom were excluded for not meeting the inclusion criteria. In the end, 216 children participated in this study, being 49,5% female (107 girls) and 50,5% male (109 boys). For better data verification, subjects were divided into three groups according to age: 4 years to 4 years and 11 months (n=77), 5 years to 5 years and 11 months (n=69), 6 years to 6 years and 11 months (n=70).

The tables presented subsequently are extensive; however, it is believed that it is essential to present the values ​​in detail, in line with the objective of this study, which is to propose standardization values ​​for the Horus® computerized posturography platform. It is worth noting that these values ​​have not yet been described and proposed for the age group researched in this specific equipment. Therefore, it is hoped that these findings can help other researchers in future studies carried out in this population.

The values ​​referring to the stability limit, according to the variables age and sex, can be seen in Table 1.

**Table 1:** Analysis of the stability limit (mm^2^) according to the variables age and sex, for the Horus® Platform (n= 216)

GROUP	N	Mean	SD	Minimum	Percentile 05	Percentile 25	Median	Percentile 75	Percentile 95	Maximum	p-value*
COMPARISON BETWEEN AGES											
4 YEARS(A)	77	8689	4048.4	1959.3	3362.5	5615.2	7745.6	11149.7	16891.2	18741.9	<0.001** A≠(B.C)
5 YEARS(B)	69	10646.4	3613.7	3168.4	5511.4	8444.3	10171.5	12409.6	16732.4	21518.2	
6 YEARS (C)	70	11342.9	4142.2	3837.6	5318.2	8064.1	11313.9	14533.7	17371.3	21182.8	
COMPARISON BY GENDER – 4 YEARS											
FEMALE	38	7540	3793.6	3115.6	3362.5	5071.3	6550.8	8623.5	17409.7	17776	0.007**
MALE	39	9808.7	4019.9	1959.3	3136.4	6616	9567.1	12481.4	16785.2	18741.9	
COMPARISON BY GENDER – 5 YEARS											
FEMALE	35	10231.6	4061.1	3168.4	3176.3	8026.5	9601.4	11997.2	21368.6	21518.2	0.275
MALE	34	11073.3	3089.7	5511.4	7398.2	8677.1	10299.2	13883.2	15914.1	18196.6	
COMPARISON BY GENDER – 6 YEARS											
FEMALE	34	10465.2	4211.5	3837.6	3931	7166.2	10298.4	13492.9	17371.3	21182.8	0.065
MALE	36	12171.9	3955.6	5437.9	5837.2	8811.1	12740.1	15540	18086	18109.5	

Caption: N = volunteers; SD = standard deviation

* P-value referring to the Kruskal-Wallis test for analysis between ages and Mann-Whitney U test for analysis between genders;

** Statistically significant result

Table 2 presents the findings obtained in the EFR evaluation, the answers are presented in the seven exam conditions, by age group.

**Table 2:** Analysis of numerical values in residual functional balance. in different exam conditions. for the Horus® Platform by age

AGE	N	VARIABLE	Mean ± SD	Minimum	Percentile 05	Percentile 25	Median	Percentile 75	Percentile 95	Maximum	p-value*
4 YEARS (A)	77	CE, C1	778.0 ± 510.8	80.6	179.7	419.5	662.1	996.5	1793.4	2610.2	<0.001** A≠C. B≠C
5 YEARS (B)	69	CE, C1	689.9 ± 755.1	82.9	151.6	323	543.8	822.8	1327.6	5731	
6 YEARS (C)	70	CE, C1	469.6 ± 366.6	69.3	126.7	227.1	393.2	599.4	1036.1	2169.1	
4 YEARS (A)	77	MLS, C1	10.1 ± 4.5	3.5	4.2	6.5	8.4	12.8	19.3	21.7	0.009** A≠C
5 YEARS (B)	69	MLS, C1	8.5 ± 3.5	2.9	4.1	6.3	8	10.2	16.6	19.5	
6 YEARS (C)	70	MLS, C1	7.8 ± 3.4	2.9	3.6	5.1	7.2	9.6	14.2	17.7	
4 YEARS (A)	77	APS, C1	15.1± 4.7	6.8	8.8	11.5	14	18.7	24.3	29.1	<0.001** A≠(B.C)
5 YEARS (B)	69	APS, C1	12.4 ± 3.9	6.2	7	10	11.9	14.1	20.7	25.2	
6 YEARS (C)	70	APS, C1	10.8 ± 3.5	5.4	5.8	7.9	11	13.1	17	21.2	
4 YEARS (A)	77	RFB, C1	90.2 ± 6.1	71.5	76.8	87.6	91	95	97.4	99	<0.001** A≠B. A≠C. B≠C
5 YEARS (B)	69	RFB, C1	93.8 ± 4.1	83.6	85.2	91.6	94.8	97	98.7	99.1	
6 YEARS (C)	70	RFB, C1	95.8 ± 2.8	84.7	90.8	94.4	96.4	97.7	98.9	99.3	
4 YEARS (A)	77	CE, C2	955.9 ± 637.3	100.5	223	454.9	811.6	1240	1957.5	3301.8	<0.001** A≠(B.C)
5 YEARS (B)	69	CE, C2	687.0 ± 536.1	68.7	172.4	375.9	560.6	783.8	1448	3959.8	
6 YEARS (C)	70	CE, C2	543.2 ± 328.5	88.4	139.5	264.4	511.3	750.3	1160.4	1725.5	
4 YEARS (A)	77	MLS, C2	12.5 ± 5.4	3.5	5.7	8.6	11.8	15.9	25.2	29.7	0.001** A≠C
5 YEARS (B)	69	MLS, C2	10.4 ± 4.1	3.5	4.3	7.8	9.8	12.6	16.8	25.6	
6 YEARS (C)	70	MLS, C2	9.5 ± 4.1	3.4	3.7	6.2	9.4	11.5	18.1	22.2	
4 YEARS (A)	77	APS, C2	21.1 ± 6.3	9.8	11.5	15.9	20.1	25	32.5	35.5	<0.001** A≠(B.C)
5 YEARS (B)	69	APS, C2	17.6 ± 5.5	8	9.6	13.7	16.8	21	27.2	36.1	
6 YEARS (C)	70	APS, C2	16.0 ± 5.8	7.2	7.9	12.5	15.6	18.8	27.7	37.3	
4 YEARS (A)	77	RFB, C2	88.4 ± 6.4	74.1	75.7	83.9	90.4	93.1	97.2	98.7	<0.001** A≠(B.C)
5 YEARS (B)	69	RFB, C2	93.1 ± 4.7	74.7	84.5	90.8	94.5	96.4	98.5	99.1	
6 YEARS (C)	70	RFB, C2	95.0 ± 3.0	81.7	90.2	93.6	95.3	97.4	98.5	99.5	
4 YEARS (A)	77	CE, C3	1036.4 ± 589.6	204.1	266.8	648.9	944.2	1385.3	2263	3219.4	<0.001** A≠(B.C)
5 YEARS (B)	69	CE, C3	798.0 ± 427.7	214.9	294.4	515.3	699	1015.5	1583.2	2612.8	
6 YEARS (C)	70	CE, C3	679.6 ± 325.5	160.6	205.9	439.1	627.6	854.4	1367.8	1562.3	
4 YEARS (A)	77	MLS, C3	14.4 ± 4.9	5.3	6.5	11	13.3	18.8	23	25.4	0.004** A≠C
5 YEARS (B)	69	MLS, C3	12.6 ± 4.1	6	7.3	9.8	12.1	15	20	27.2	
6 YEARS (C)	70	MLS, C3	11.9 ± 4.2	5.5	6.5	8.6	11.1	14	19.9	22.9	
4 YEARS (A)	77	APS, C3	19.43 ± 5.26	7.5	11.2	16.1	18.6	22.3	29.4	34.7	<0.001** A≠(B.C)
5 YEARS (B)	69	APS, C3	16.84 ± 4.95	9.6	10.7	12.8	15.4	20	25.7	31.1	
6 YEARS (C)	70	APS, C3	15.70 ± 5.03	8.6	9.5	12.3	15.1	17.5	24.8	38.7	
4 YEARS (A)	77	RFB, C3	86.9 ± 7.3	49.9	73.7	83.2	88.4	91.6	96.2	98.2	<0.001** A≠(B.C)
5 YEARS (B)	69	RFB, C3	91.9 ± 4.2	74.5	84.9	89.8	92.4	95.1	97.2	97.8	
6 YEARS (C)	70	RFB, C3	93.5 ± 3.2	80.4	87.8	92.1	93.9	95.4	97.8	98.4	
4 YEARS (A)	77	CE, C4	1530.9 ±661.5	438	639.2	998.7	1522.1	1979.2	2815	3500.9	0.007** A≠(B.C)
5 YEARS (B)	69	CE, C4	1317.6 ±718.2	457.7	529.1	898.8	1134.2	1541.8	2770	4693.8	
6 YEARS (C)	70	CE, C4	1220.7 ±574.1	369.5	468	765.4	1227.4	1523.4	2432.1	3016.9	
4 YEARS (A)	77	MLS, C4	21.1 ± 7.4	8.5	11.7	15.8	19.6	24.4	38.9	43.6	0.404
5 YEARS (B)	69	MLS, C4	19.6 ± 6.9	8	9.3	15.3	18.2	23.1	32.6	37.7	
6 YEARS (C)	70	MLS, C4	19.8 ± 7.1	8.6	10.6	13.6	18.9	24	32.4	44.9	
4 YEARS (A)	77	APS, C4	31.3 ± 7.9	15.4	18.1	26.3	30.4	36.6	44.1	52.8	<0.001** A≠(B.C)
5 YEARS (B)	69	APS, C4	27.4 ± 8.6	13.8	14.8	21.1	26.4	31.4	42.8	52.2	
6 YEARS (C)	70	APS, C4	26.3 ± 8.9	13.4	15	19.6	24.5	30.5	48.7	51.2	
4 YEARS (A)	77	RFB, C4	80.6 ± 8.1	52.4	66.6	75.5	82	86	91.2	95.7	<0.001** A≠(B.C)
5 YEARS (B)	69	RFB, C4	86.6 ± 7.9	49.1	73.9	81.9	88.1	91.9	95.4	96.5	
6 YEARS (C)	70	RFB, C4	88.6 ± 4.9	72.2	81.4	85.2	89	91.3	96.4	97	
4 YEARS (A)	77	CE, C5	1137.0 ±531.6	385	452.7	714.2	1059.8	1425.3	2029.4	2810.9	0.028** A≠C
5 YEARS (B)	69	CE, C5	1039.0 ±614.0	371	423.4	679.2	913.7	1220.1	2088.6	4294	
6 YEARS (C)	70	CE, C5	896.9 ± 399.2	207.1	379.3	600.3	872.9	1154.9	1615.2	2311.6	
4 YEARS (A)	77	MLS, C5	15.9 ± 5.0	6.5	8.7	12.1	15.2	19.1	24.1	35	0.727 A≠C
5 YEARS (B)	69	MLS, C5	15.4 ± 4.8	7	9.4	12.6	14.5	17.4	25.7	33.7	
6 YEARS (C)	70	MLS, C5	15.5 ± 5.5	6.5	7.7	10.8	15	18.6	24.9	29.9	
4 YEARS (A)	77	APS, C5	21.3 ± 4.9	11.6	13.5	17.5	21.4	25.3	30.4	32.6	0.14
5 YEARS (B)	69	APS, C5	20.4 ± 5.1	11.5	12.8	17.3	19.5	23.4	30.7	34.9	
6 YEARS (C)	70	APS, C5	19.8 ± 6.0	10.4	11.4	15.6	19.4	23.7	30.3	43.3	
4 YEARS (A)	77	RFB, C5	85.7 ± 6.0	66.1	74.6	82.3	86	90.1	93.6	95.7	<0.001** A≠(B.C)
5 YEARS (B)	69	RFB, C5	89.7 ± 5.3	72.6	80.3	87.6	90.6	93.9	95.9	96.1	
6 YEARS (C)	70	RFB, C5	91.5 ± 3.5	82.5	85.3	89.2	92	94.1	96.1	98	
4 YEARS (A)	77	CE, C6	1199.9± 639.6	347.8	518.9	714.9	1148.6	1502.2	2603.1	3715.5	0.049**
5 YEARS (B)	69	CE, C6	1012.4± 612.7	270.8	410.9	692	899.7	1270.1	1938.1	4195.4	
6 YEARS (C)	70	CE, C6	6936.7± 392.3	173.5	291.5	661.3	949.2	1198.7	1545.1	2056.3	
4 YEARS (A)	77	MLS, C6	16.3 ± 5.4	8	8.4	12.4	15.4	19.4	27.4	30.4	0.666
5 YEARS (B)	69	MLS, C6	15.7 ± 5.6	7.3	8.7	12	14.6	18.5	24.7	35.2	
6 YEARS (C)	70	MLS, C6	15.5 ± 5.4	5.3	8.8	12	14.7	18.8	24.2	32.3	
4 YEARS (A)	77	APS, C6	21.7 ± 5.8	13.4	14.5	17.7	19.8	25.5	33.2	38.4	0.028** A≠C
5 YEARS (B)	69	APS, C6	21.0 ± 10.1	10.9	12.6	16.2	19.1	22.4	32.5	92.3	
6 YEARS (C)	70	APS, C6	19.2 ± 5.5	10.1	12.7	15.1	18.5	21.7	30.2	36.4	
4 YEARS (A)	77	RFB, C6	84.9 ± 7.0	55.9	73.1	81.9	86	89.8	94.2	96.5	<0.001** A≠(B.C)
5 YEARS (B)	69	RFB, C6	89.8 ± 5.6	72.5	80.3	86.6	91.2	94.1	96.2	97.3	
6 YEARS (C)	70	RFB, C6	91.0 ± 4.0	79.5	83.4	88.3	91.7	94	97.2	98	
4 YEARS (A)	77	CE, C7	1098.8 ± 478.7	355.6	480.6	741.4	1073	1371.5	2004.9	2513.1	0.001** A≠C. B≠C
5 YEARS (B)	69	CE, C7	1016.0 ± 535.2	343.4	423.6	690.2	905.6	1188	1969	3747.5	
6 YEARS (C)	70	CE, C7	811.0 ± 383.6	271.7	304.5	562.4	747	1030.9	1428	1973.8	
4 YEARS (A)	77	MLS, C7	13.9 ± 3.6	6.7	8.3	11.4	13.5	16.2	21.2	22.1	0.115
5 YEARS (B)	69	MLS, C7	13.3 ± 3.8	6	8.2	10.7	12.8	15.4	19.4	24.7	
6 YEARS (C)	70	MLS, C7	12.7 ± 3.8	4.9	7.8	9.9	12.3	15.3	18.5	24.2	
4 YEARS (A)	77	APS, C7	21.5 ± 4.5	12.4	14.5	18.5	21.6	24.8	30	31.2	<0.001** A≠C
5 YEARS (B)	69	APS, C7	20.0 ± 4.7	10.4	13.4	17	18.9	22.8	28.3	31.1	
6 YEARS (C)	70	APS, C7	18.4 ± 5.3	10.1	11.3	14.6	17.7	20.6	27	39.6	
4 YEARS (A)	77	RFB, C7	85.7 ± 6.9	61.7	73.3	82.6	87.3	91.1	93.6	94.6	<0.001** A≠(B.C)
5 YEARS (B)	69	RFB, C7	89.7 ± 58.9	67.9	76.1	87.4	91.1	93.9	95.9	96.7	
6 YEARS (C)	70	RFB, C7	91.9 ± 4.4	80.3	82.8	90.1	92.9	95.2	97.5	98.1	

Caption: N = volunteers; SD = standard deviation; CE = Confidence Ellipse; MLS = Medial-Lateral speed; APS = anteroposterior speed; RFB = residual functional balance; C1 = eyes open condition. stable surface (mm^2^); C2 = eyes closed condition. stable surface (mm^2^); C3 = eyes open condition. unstable surface (mm^2^); C4 = eyes closed condition. unstable surface (mm^2^); C5 = optokinetic stimulation condition to the right unstable surface (mm^2^); C6 = optokinetic stimulation condition to the left. unstable surface (mm^2^); C7 = unstable surface. tunnel stimulation condition (mm^2^)

* P-value referring to the Kruskal-Wallis test for comparison of values between 3 groups and Dunn-Bonferroni post hoc test for pairwise comparison across ages

Table 3 presents the findings that showed statistically significant differences in the assessment of functional balance, by age and sex, in the seven exam conditions.

**Table 3:** Statistically significant results in residual functional balance, by age, in different exam conditions, for the Horus® Platform when compared by the gender variable

AGE AND GENDER	N	VARIABLE	Mean ± SD	Minimum	Percentile 05	Percentile 25	Median	Percentile 75	Percentile 95	Maximum	p-value*
4 YEARS, F	38	CE, C1	617.3 ± 412.5	80.6	131.0	309.0	549.8	738.0	1715.2	1780.7	0.004
4 YEARS, M	39	CE, C1	934.6 ± 552.5	118.4	240.5	537.8	814.3	1299.4	1913.4	2610.2	
5 YEARS, F	35	CE, C1	498.1 ± 320.1	82.9	105.8	192.9	444.8	685.3	1062.2	1295.3	0.019
5 YEARS, M	34	CE, C1	887.2 ± 995.2	181.5	204.6	448.9	631.5	887.2	2428.1	5731.0	
4 YEARS, F	38	MLS, C1	8.7 ± 3.6	3.5	4.0	6.0	7.5	12.4	15.0	15.4	0.012
4 YEARS, M	39	MLS, C1	11.4 ± 4.9	4.2	4.5	7.4	11.3	14.7	20.2	21.7	
5 YEARS, F	35	MLS, C1	7.5 ± 2.5	2.9	3.1	5.8	7.2	9.0	12.5	13.0	0.05
5 YEARS, M	34	MLS, C1	9.6 ± 4.1	4.1	4.3	7.0	8.5	10.8	19.4	19.5	
6 YEARS, F	34	MLS, C1	7.0 ± 3.3	3.3	3.6	4.7	6.1	8.3	14.4	17.5	0.019
6 YEARS, M	36	MLS, C1	8.5 ± 3.2	2.9	3.2	6.7	8.3	10.5	14.2	17.7	
4 YEARS, F	38	APS, C1	13.9 ± 4.5	6.8	8.5	10.0	12.9	17.4	22.3	24.9	0.016
4 YEARS, M	39	APS, C1	16.4 ± 4.6	9.6	10.2	12.9	15.7	19.5	24.8	29.1	
5 YEARS, F	35	APS, C1	11.3 ± 3.4	6.2	6.3	9.3	10.4	13.4	17.1	20.7	0.007
5 YEARS, M	34	APS, C1	13.6 ± 4.0	7.0	7.2	11.3	13.3	15.3	21.5	25.2	
6 YEARS, F	34	APS, C1	9.8 ± 3.3	5.4	5.5	7.6	9.1	11.9	14.6	21.2	0.011
6 YEARS, M	36	APS, C1	11.8 ± 3.3	6.0	6.1	9.7	11.9	13.7	17.5	19.6	
4 YEARS, F	38	CE, C2	787.2 ± 588.1	100.5	168.0	412.2	602.3	1036.1	1889.2	2784.4	0.007
4 YEARS, M	39	CE, C2	1120.4 ± 647.5	223.0	248.1	659.4	1045.3	1575.0	2319.4	3301.8	
6 YEARS, F	34	CE, C2	442.0 ± 264.3	88.4	127.8	220.8	364.4	619.7	940.0	1175.8	0.017
6 YEARS, M	36	CE, C2	638.7 ± 357.2	138.1	139.5	326.1	656.1	872.9	1225.5	1725.5	
5 YEARS, F	35	MLS, C2	9.1 ± 3.0	3.5	3.8	7.2	9.2	11.5	14.3	14.7	0.027
5 YEARS, M	34	MLS, C2	11.6 ± 4.6	5.1	6.2	8.1	11.4	13.9	22.5	25.6	
6 YEARS, F	34	MLS, C2	8.2 ± 3.8	3.4	3.5	5.8	7.4	9.8	16.3	20.4	0.004
6 YEARS, M	36	MLS, C2	10.7 ± 4.1	3.4	3.7	8.5	10.5	12.6	19.2	22.2	
4 YEARS, F	38	APS, C2	19.6 ± 6.8	9.8	10.0	14.2	18.1	23.3	34.2	35.5	0.021
4 YEARS, M	39	APS, C2	22.5 ± 5.5	13.7	14.4	18.2	22.4	25.5	32.5	33.2	
5 YEARS, F	35	APS, C2	16.2 ± 5.0	8.0	8.9	12.2	16.1	19.1	25.1	29.2	0.044
5 YEARS, M	34	APS, C2	19.0 ± 5.6	12.6	13.3	14.6	17.8	22.4	32.7	36.1	
6 YEARS, F	34	APS, C2	13.6 ± 4.2	7.2	7.4	10.8	13.3	16.1	22.2	22.9	>0.001
6 YEARS, M	36	APS, C2	18.2 ± 6.2	9.0	9.1	15.1	16.9	20.6	33.9	37.3	
4 YEARS, F	38	CE, C3	844.7 ± 455.7	204.1	233.8	466.6	762.9	1076.4	1635.5	1855.6	0.005
4 YEARS, M	39	CE, C3	1223.1±648.4	233.2	299.0	823.5	1133.4	1504.6	3079.4	3219.4	
5 YEARS, F	35	CE, C3	637.8 ± 261.8	214.9	252.6	445.2	612.4	800.8	1080.9	1139.3	0.001
5 YEARS, M	34	CE, C3	-5.5 ± 9.2	-25.5	-22.7	-9.3	-5.7	.9	9.1	9.9	
4 YEARS, F	38	MLS, C3	12.8 ± 5.1	5.3	5.6	10.1	12.3	14.1	23.1	25.4	0.003
4 YEARS, M	39	MLS, C3	15.9 ± 4.3	8.5	8.6	12.0	14.9	19.1	22.7	24.5	
5 YEARS, F	35	MLS, C3	11.3 ± 2.9	6.0	7.3	9.4	11.2	13.8	15.7	18.5	0.015
5 YEARS, M	34	MLS, C3	13.9 ± 4.8	6.0	6.6	10.4	13.1	15.9	23.8	27.2	
6 YEARS, F	34	MLS, C3	10.6 ± 4.0	5.5	6.1	8.0	9.4	12.2	19.1	19.9	0.003
6 YEARS, M	36	MLS, C3	13.2 ± 4.1	5.5	7.4	10.8	12.7	15.8	21.6	22.9	
4 YEARS, F	38	APS, C3	18.19 ± 5.51	7.50	9.90	15.00	17.05	21.00	30.50	31.70	0.017
4 YEARS, M	39	APS, C3	20.64 ± 4.77	12.00	12.60	17.60	20.90	23.20	28.80	34.70	
5 YEARS, F	35	APS, C3	15.39 ± 4.31	9.60	9.70	12.80	13.90	16.30	24.90	27.30	0.009
5 YEARS, M	34	APS, C3	18.35 ± 5.18	10.60	10.70	15.00	17.60	20.90	31.00	31.10	
6 YEARS, F	34	APS, C3	14.53 ± 5.55	9.40	9.50	11.10	13.40	16.30	26.20	38.70	0.004
6 YEARS, M	36	APS, C3	16.80 ± 4.27	8.60	8.80	13.95	16.75	19.00	24.80	26.30	
5 YEARS, F	35	RFB, C3	93.1 ± 3.2	84.8	87.0	90.2	93.8	95.5	97.6	97.8	0.027
5 YEARS, M	34	RFB, C3	90.7 ± 4.8	74.5	79.9	88.3	91.4	94.2	97.0	97.2	
4 YEARS, F	38	CE, C4	1308.8 ± 636.7	438.0	469.7	863.5	1058.6	1712.4	2815.0	3195.3	0.002
4 YEARS, M	39	CE, C4	1747.3 ± 618.9	721.3	729.3	1319.6	1757.6	2124.3	3019.3	3500.9	
6 YEARS, F	34	CE, C4	1018.9 ± 515.1	369.5	438.0	608.4	942.0	1279.0	2030.3	2612.3	0.001
6 YEARS, M	36	CE, C4	1411.3 ± 568.0	468.0	524.6	1091.8	1343.1	1662.8	2847.2	3016.9	
6 YEARS, F	34	MLS, C4	16.7 ± 5.5	8.6	9.9	12.4	16.1	19.9	28.0	29.6	<0.001
6 YEARS, M	36	MLS, C4	22.9 ± 7.2	12.5	12.5	18.6	22.0	26.1	33.6	44.9	
4 YEARS, F	38	APS, C4	29.3 ± 7.1	15.4	16.0	25.3	27.7	31.9	43.5	46.7	0.039
4 YEARS, M	39	APS, C4	33.2 ± 8.3	17.8	20.0	26.8	32.2	40.6	46.3	52.8	
5 YEARS, F	35	APS, C4	25.4 ± 8.7	13.8	13.9	18.9	25.1	28.9	44.1	47.7	0.028
5 YEARS, M	34	APS, C4	29.4 ± 8.0	15.5	18.2	22.7	29.7	34.8	42.3	52.2	
6 YEARS, F	34	APS, C4	22.4 ± 5.3	13.4	14.1	18.5	21.7	25.5	33.4	34.0	0.001
6 YEARS, M	36	APS, C4	30.0 ± 10.0	14.9	16.4	23.3	28.7	34.0	49.2	51.2	
4 YEARS, F	38	CE, C5	919.2 ± 421.2	385.0	395.3	594.7	795.7	1167.0	1840.0	1914.1	<0.001
4 YEARS, M	39	CE, C5	1349.2 ± 546.6	540.5	572.9	905.6	1320.8	1800.9	2462.0	2810.9	
5 YEARS, F	35	CE, C5	876.5 ± 371.6	389.9	415.6	545.0	786.3	1143.9	1606.9	1620.0	0.033
5 YEARS, M	34	CE, C5	1206.2 ± 760.0	371.0	478.1	795.8	1056.7	1264.2	2758.2	4294.0	
6 YEARS, F	34	CE, C5	735.6 ± 289.1	207.1	250.3	523.1	705.7	949.3	1247.7	1289.9	0.002
6 YEARS, M	36	CE, C5	1049.2 ± 431.5	377.3	389.1	787.6	962.2	1343.5	1808.2	2311.6	
4 YEARS, F	38	MLS, C5	14.4 ± 4.7	6.5	7.8	11.3	13.1	18.4	23.7	26.1	0.01
4 YEARS, M	39	MLS, C5	17.3 ± 4.9	9.9	10.1	12.8	17.5	19.6	24.2	35.0	
6 YEARS, F	34	MLS, C5	13.4 ± 4.2	6.5	7.4	10.0	12.9	17.0	19.8	24.6	0.005
6 YEARS, M	36	MLS, C5	17.4 ± 5.9	6.8	8.1	12.2	17.3	21.3	28.3	29.9	
4 YEARS, F	38	APS, C5	20.1 ± 4.8	11.6	12.6	16.6	19.1	22.4	30.4	31.1	0.033
4 YEARS, M	39	APS, C5	22.4 ± 4.7	13.5	13.8	18.5	22.6	26.0	30.7	32.6	
6 YEARS, F	34	APS, C5	18.2 ± 4.4	10.4	11.6	15.3	18.2	21.1	26.1	30.3	0.04
6 YEARS, M	36	APS, C5	21.4 ± 6.8	11.3	11.3	17.6	20.0	25.0	35.6	43.3	
4 YEARS, F	38	CE, C6	955.1 ± 443.5	427.4	452.1	602.3	842.0	1185.4	2091.9	2385.1	0.001
4 YEARS, M	39	CE, C6	1438.4 ± 713.0	347.8	537.6	884.6	1223.4	1735.8	2857.8	3715.5	
4 YEARS, F	38	MLS, C6	15.1 ± 5.4	8.0	8.3	10.9	14.0	18.4	27.4	30.4	0.034
4 YEARS, M	39	MLS, C6	17.4 ± 5.1	9.7	10.6	13.7	16.0	20.9	29.6	29.9	
6 YEARS, F	34	MLS, C6	14.2 ± 5.0	5.3	7.1	10.6	14.2	15.8	27.4	30.0	0.042
6 YEARS, M	36	MLS, C6	16.7 ± 5.5	7.4	9.0	12.8	16.0	20.8	24.2	32.3	
4 YEARS, F	38	APS, C6	19.9 ± 4.5	13.4	13.5	16.3	18.8	23.6	28.6	28.7	0.015
4 YEARS, M	39	APS, C6	23.4 ± 6.5	13.6	14.5	18.2	22.2	27.4	37.8	38.4	
6 YEARS, F	34	APS, C6	17.9 ± 5.3	10.1	12.7	14.1	16.6	19.4	29.5	34.4	0.013
6 YEARS, M	36	APS, C6	20.5 ± 5.4	10.2	12.6	17.1	20.7	23.1	30.3	36.4	
4 YEARS, F	38	CE, C7	987.3 ± 455.2	355.6	399.7	547.9	906.2	1307.6	1972.0	2004.9	0.036
4 YEARS, M	39	CE, C7	1207.5 ± 481.6	493.1	520.7	817.3	1153.2	1451.6	2164.5	2513.1	
6 YEARS, F	34	CE, C7	691.9 ± 316.1	271.7	280.1	479.6	622.1	836.5	1370.0	1410.7	0.012
6 YEARS, M	36	CE, C7	923.5 ± 411.2	381.4	387.3	604.1	825.9	1197.0	1842.4	1973.8	
4 YEARS, F	38	MLS, C7	12.8 ± 3.7	6.7	6.7	9.8	13.0	15.0	21.2	21.4	0.026
4 YEARS, M	39	MLS, C7	14.9 ± 3.3	9.6	10.0	12.0	14.1	17.9	21.2	22.1	
6 YEARS, F	34	MLS, C7	11.1 ± 2.9	4.9	7.0	9.2	10.2	13.0	16.5	17.9	0.001
6 YEARS, M	36	MLS, C7	14.1 ± 4.0	5.1	8.6	11.0	13.7	16.8	21.6	24.2	
6 YEARS, F	34	APS, C7	16.6 ± 3.9	10.1	10.8	14.1	16.1	19.6	24.5	25.6	0.009
6 YEARS, M	36	APS, C7	20.0 ± 6.0	10.4	12.7	16.0	19.5	20.9	34.4	39.6	

Caption: N = volunteers; SD = standard deviation; F = Female; M = Male; CE = Confidence Ellipse; MLS = Medial-Lateral speed; APS = anteroposterior speed; RFB = residual functional balance; C1 = eyes open condition, stable surface (mm^2^); C2 = eyes closed condition, stable surface (mm^2^); C3 = eyes open condition, unstable surface (mm^2^); C4 = eyes closed condition, unstable surface (mm^2^); C5 = optokinetic stimulation condition to the right, unstable surface (mm^2^); C6 = optokinetic stimulation condition to the left, unstable surface (mm^2^); C7 = unstable surface, tunnel stimulation condition (mm^2^)

* P-value referring to the U de Mann-Whitney test

Finally, the analysis of responses in sensory systems by age group is presented in Table 4.

**Table 4:** Sensory analysis in comparison by age group, in mm^2^, for the Horus® Platform (n= 216)

AGE	SYSTEM	N	Mean	SD	Minimum	Percentile 05	Percentile 25	Median	Percentile 75	Percentile 95	Maximum	p-value*
4 YEARS (A)	SOM	77	98.1	5.1	82.4	86.7	95.7	97.7	100.4	106.3	109.8	0.184
5 YEARS (B)	SOM	69	99.1	3.5	87.7	94.3	97.3	99.1	101.1	103.8	111.8	
6 YEARS (C)	SOM	70	99.2	2.8	92.5	94.8	97.8	99.1	100.3	102.6	111.8	
4 YEARS (A)	VIS	77	96.5	5.5	68.6	88.5	94.0	96.3	99.4	104.8	107.2	0.089
5 YEARS (B)	VIS	69	98.1	3.8	85.1	92.3	96.2	98.2	100.0	103.4	113.0	
6 YEARS (C)	VIS	70	97.6	2.7	88.5	93.3	96.2	98.2	99.1	101.3	105.3	
4 YEARS (A)	VEST	77	89.3	7.6	71.6	76.7	84.6	88.8	95.2	101.5	106.2	0.006**A≠(B.C))
5 YEARS (B)	VEST	69	92.2	7.3	54.6	83.3	89.0	92.9	96.6	100.8	109.6	
6 YEARS (C)	VEST	70	92.5	4.8	74.8	85.2	89.9	93.1	95.3	98.1	110.2	
4 YEARS (A)	VDPR	77	105.8	13.7	11.8	94.9	100.1	106.4	112.0	125.0	127.2	0.024** A≠B
5 YEARS (B)	VDPR	69	104.1	7.8	93.8	95.9	99.6	102.6	106.9	116.1	151.7	
6 YEARS (C)	VDPR	70	103.5	4.1	95.8	97.9	100.5	103.4	106.3	111.1	114.3	
4 YEARS (A)	VDPL	77	105.6	9.4	60.4	93.9	99.6	105.7	110.9	121.2	124.6	0.033** A≠C
5 YEARS (B)	VDPL	69	104.2	7.9	95.1	96.0	100.3	102.4	106.5	115.0	153.0	
6 YEARS (C)	VDPL	70	101.6	11.9	11.5	96.0	99.9	102.5	104.7	110.9	121.9	
4 YEARS (A)	VDPT	77	107.4	7.5	96.1	97.3	102.0	105.8	111.8	122.4	131.5	0.002** A≠(B.C)
5 YEARS (B)	VDPT	69	102.6	12.9	11.8	96.0	99.9	101.7	106.6	113.7	138.3	
6 YEARS (C)	VDPT	70	102.7	12.3	11.5	95.6	100.9	104.0	107.3	111.4	124.0	
4 YEARS (A)	CBI	77	85.4	5.9	59.2	73.0	82.1	87.0	88.9	92.4	96.3	<0.001** A≠(B.C)
5 YEARS (B)	CBI	69	90.0	4.9	73.6	80.3	88.0	90.8	93.8	95.8	96.9	
6 YEARS (C)	CBI	70	91.8	3.1	83.1	86.2	89.9	92.2	93.6	97.0	97.5	

Caption: N = volunteers; SD = standard deviation; SOM = somatosensory (%); VIS = visual (%); VEST = vestibular (%); VDPR = visual optokinetic dependence to the right (%); VDPL = visual optokinetic dependence to the left (%); VDPT = visual tunnel dependence (%); CBI = composite balance index (%)

* P-value referring to the Kruskal-Wallis test;

** Statistically significant result

Other important findings refer to the analysis of sensory responses by sex at the ages of 4 and 5 years, it was found that there was no statistically significant difference between boys and girls. Whereas, in children aged 6 years, there was a difference only for the stages related to the visual system. Significant findings in left dependence (p=0.009), in which boys presented higher values ​​for both the mean, median and minimum value (percentiles 05, 25, 75, and 95), only for the left dependence situation and, in the tunnel situation (p=0.04) with lower values ​​compared to girls for maximum value and 95th percentile.

## DISCUSSION

The static posturography platform with dynamic tests Horus® and its software were developed by a Brazilian company to meet the demands of healthcare professionals. The company aims to provide a portable and user-friendly device, intending to reduce costs. Previously, posturographic equipment available in the market was imported and came with high costs. The platform in question was officially launched in 2017 and has been the focus of studies^(9-11,18)^, as it allows the assessment of body balance and rehabilitation through games^(19)^. However, in the child population, there are still no studies showing its use and normative values^(7)^.

Using dynamic posturography with virtual reality, Balance Rehabilitation Unit -BRU™, a study analyzed the responses of children aged 7 to 12 years, separated into groups with and without school difficulties, highlighting the limitation in carrying out data analysis, due to the lack of of standardization for children^(12)^. However, in the research it was possible to observe differences between groups for speed of oscillation and area of displacement of the pressure center^(12)^. Researchers believe that for the adequate analysis of posturography results, it is important to standardize the data selected and considered, since the statokinesigram and stabilogram generate variables that are often redundant and unnecessary to the examiner, thus, they suggested the use of the main variables referring to the area and total average velocity in both directions, anteroposterior and mediolateral, in relation to the PC^(2)^.

A different study conducted using the dynamic posturography platform Foam-Laser in children aged 6 to 10 years demonstrated that regardless of sex, children present lower responses than adults, highlighting the effect of maturation of body balance^(20)^. The findings of the present study, on static type equipment, demonstrated a change in mean values with increasing age for CE and anteroposterior and mediolateral velocity data (Table 2) in the seven examination conditions, with a reduction in values with increasing age, indicative of gradual evolution in the maturation of body balance;. Which is in line with the study mentioned previously^(20)^, that despite dealing with different age groups, the researchers demonstrated the effect of maturation on postural control by comparing responses between children and adults. This reinforces the need to perform data analysis separately according to age group, as well as the importance of standardizing posturography responses in children aged 4 to 6 years old.

According to Ferreira et al.^(10)^ and Nishino et al.^(11)^ in the analysis of responses in adults, using the same posturography equipment used in the present study, Horus®, the analysis of the stability limit must be considered distinct by sex and age group, as males obtained responses with higher values than females. Our findings partially corroborate this, as we observed an intra-group difference by sex only for the age of 4 years (Table 1), with the values being higher in boys. It is believed that this finding may be related to the difference in the height of the subjects, as boys are higher, on average, only in this age group.

In the analysis of the answers of children aged 3 to 6 years old in the Equitest® dynamic posturography exam, researchers found a statistically significant difference when comparing responses between the 3 and 4 year olds with the 5 and 6 year olds, being the smallest less stable^(21)^. The present research, carried out in the studied age range of 4 to 6 years, corroborates the researchers, even using static posturography, as the values related to body oscillation and displacement were higher in younger subjects. Furthermore, the responses observed in the RFB increased the percentage value as the age increased, in the seven examination conditions (Table 2). Others researchers assert that body stability is represented by RFB data in posturography, which can be observed quantitatively and providing the area still available for the subject's sway^(18)^. The closer to 100% the greater the patient's body stability in the examination condition evaluated^(18)^.

According to researchers, the maturation of the sensory systems involved with body balance, occurs respecting the following order: visual system, followed by the proprioceptive system and, finally, it occurs in the vestibular system, reaching functional maturity at nine years of age^(22)^. Other research concluded that the transition to responses similar to the adult pattern will not be complete at the age of 6, for all sensory conditions^(21)^. In the results of the present research (Table 2), in the evaluation conditions where there was a demand for visual dependence, there was a significant difference between ages, with a reduction in mean values as age increased, and the opposite occurred for the RFB.

The change in responses with increasing age was observed in research using the Wii Balance Board platform and a analysis software created for the study itself, in 38 children aged 3 to 14 years^(13)^. The research indicated a significant trend towards decreased values in standing postural balance examination with eyes open up to 8 years of age, as the variation in the center of pressure decreased significantly with increasing age of the child.^(13)^. Statistically, this finding was not frequent in all examination conditions in the comparison between ages (Table 2), possibly because it is a different posturography platform than the one used in the present study.

The same authors mentioned above reported that, when verifying the responses in the test conditions with eyes open and eyes closed, regardless of age, the values of postural balance increased significantly when the child had his/her eyes closed^(13)^. When observing the results comparing examination conditions C1 with C2 and C3 with C4 (Table 2), an increase in mean values can be seen when the child was in examination conditions without visual support. Despite this difference was subtle, reinforcement of the statement that the lack of visual support, in young children, worsens postural control.

In the evaluation of children aged 3 to 6 years, it was observed that for both condition C1 and condition C2, eyes open and eyes closed, respectively, postural stability increased with age^(21)^. Regarding analysis by sex and responses verified by systems, using the AMTI AccuSway Plus ACS force platform, it was found that the visual system is used differently between girls and boys, in children and adolescents^(23)^. Another study shows that, in addition to the maturational issue, the control of body stability in girls has its own rhythm of development, at the fixed age of 11 to 13 years old, thus demonstrating that they are in a phase of individual development in adolescence^(24)^. In the present study, there was a difference in the responses between boys and girls (Table 3), where girls demonstrated lower values for displacement speed in relation to the PC, being more stable, as well as a lower value for the CE, corresponding to the area movement of the body without oscillation, which reinforces the importance of standardizing responses in assessing body balance in children, for adequate diagnosis, treatment, and also for use in monitoring development and/or therapeutic effectiveness.

The control of body balance changes with age, changing from primarily visual-vestibular to somatosensory-vestibular, according to study using computerized dynamic posturography^(21)^. It was observed that, for static posturography Horus®, in the findings of the current research, there was a reduction in the standard deviation value in each test condition for sensory measurements (Table 4), with increasing age, demonstrating less dispersion of values and, thus, maturational effect. It is known that the motor development of children progresses gradually, as confirmed by the results found. The Horus® posturography has been shown to be effective in determining the evolution of balance in young children, without vestibular/auditory complaints, and can, therefore, assist in the diagnosis of children with balance changes.

In children, it is noticed that up to 4 years of age they provide reevaluated multisensory information and, after 4 years of age, an intermodal response occurs, characterized as the adaptive fusion of two sensory conditions, where it was found that the postural response for one condition was dependent on the amplitude of the other condition^(25)^. Researchers report that complete interdependence of sensory systems occurs at different periods, with the somatosensory system being considered the one that develops earlier, being similar to adults, at ages between 3 and 4 years^(15,26)^, with maturation being later for the visual and vestibular systems^(15)^. The current findings show the absence of statistical difference for the visual and somatosensory systems (Table 4), in the studied range of 4 to 6 years and 11 months in body balance for the Horus® static platform, demonstrating the presence, also, of dependence on these systems for body balance at this age.

The difficulty in finding studies that have used static posturography in children generated the need for comparison with dynamic posturography during the discussion. This factor was considered one of the limitations of the study. Furthermore, the scarcity of studies for the Horus® platform is highlighted, as it is new equipment on the market. Based on the proposed values, it is suggested that further research can be carried out with the aim of investigating responses in children with atypical development and/or vestibular complaints.

## CONCLUSION

It was possible to establish normative values for the Horus® static posturography in healthy children aged 4 to 6 years. Additionally, it is suggested that, for this population and assessment tool, the responses obtained in the assessment of children should be analyzed by age group and sex.

The Horus® computerized posturography proved to be applicable and effective for determining the evolution of body balance in young children, aged 4 to 6 years and 11 months, with typical development and without vestibular and/or auditory complaints. Additionally, it can help in the diagnosis of children with alterations in body balance.
