# Supplementary material for: Normative values to Horus® computerized posturography in children
Source: Codas. 2024 Aug 5;36(5):e20230241. doi: 10.1590/2317-1782/20242023241en (PMC11340877; doi:10.1590/2317-1782/20242023241en)
Supplement: Translation

# Translation: Valores normativos para a posturografia computadorizada Horus® em crianças

**DOI:** 10.1590/2317-1782/20242023241pt

**Authors:** Ândrea de Melo Boaz, Rudimar dos Santos Riesgo, Pricila Sleifer

**Affiliations:** 1 Universidade Federal do Rio Grande do Sul – UFRGS - Porto Alegre (RS), Brasil.

**Keywords:** Equilíbrio Postural, Criança, Propriocepção, Padrões de Referência, Estudo de Validação, Crescimento e Desenvolvimento, Sistema Vestibular, Equipamentos para Diagnóstico

## Abstract

**Objetivo:**

Propor valores de normatização da plataforma de posturografia computadorizada Horus®, em crianças de 4 a 6 anos, sem queixas auditivas e/ou vestibulares.

**Método:**

Estudo transversal. Participaram 216 crianças na faixa etária de 4 a 6 anos. Todas realizaram triagem visual, avaliação auditiva e posturografia computadorizada composta por pesquisa do limite de estabilidade e sete condições sensoriais. Analisaram-se os resultados estatisticamente por testes não paramétrico Kruskal-Walli, post hoc Dunn-Bonferroni para comparações par-a-par nas idades e U de Mann-Whitney para análise entre sexo. Os dados categóricos foram apresentados em frequência relativa e os dados quantitativos pela média e desvio padrão.

**Resultados:**

Foram descritos valores de normatização para o limite de estabilidade e para as sete condições sensoriais. Houve diferença para o limite de estabilidade entre sexos aos 4 anos (p<0,007) e, na comparação entre as idades 4 e 5 anos (p=0,005) e 4 e 6 anos (p<0,001). No equilíbrio funcional residual, comparação entre idades, houve diferença entre 4 e 5, 4 e 6 e, 5 e 6 anos, entretanto para diferentes dados. A presença de diferença estatística para diferentes dados da avaliação, ocorreu também na análise por sexo. Nos sistemas sensoriais os achados entre idades mostraram diferença para o sistema vestibular, dependência visual optocinética direita e esquerda, dependência visual túnel e para índice de equilíbrio composto. Sugere-se que para esta população, as respostas na posturografia sejam analisadas por faixa etária e sexo.

**Conclusão:**

Foi possível estabelecer valores normativos para a posturografia Horus® em crianças hígidas na faixa etária de 4 a 6 anos.

## INTRODUÇÃO

A avaliação do equilíbrio corporal pode ser realizada por abordagem funcional, conforme seleção mais adequada no momento da avaliação, a critério do examinador, por meio de sistemas pela análise da biomecânica, coordenação motora e organização sensorial, ou por meio de posturografias^(1)^, de forma qualitativa, observacional ou quantitativa, com o uso de instrumentos de medição^(2)^.

Geralmente, utiliza-se a posturografia para complementar a avaliação do equilíbrio corporal, juntamente com a realização de outros procedimentos como o exame de vectoeletronistagmografia^(3)^. Isso ocorre porque estes exames têm objetivos diferentes, e a posturografia permite a avaliação da oscilação do corpo em diferentes condições de teste, gerando respostas dos sistemas visual, vestibular e somatossensorial, importantes na manutenção do equilíbrio corporal^(3,4)^. Deste modo, as respostas obtidas com o exame de posturografia permitem a percepção de como está a habilidade funcional, sendo um instrumento útil para a compreensão do uso adequado das informações do sistema vestibular pelo sujeito^(4)^.

Na população infantil, a avaliação do sistema vestibular se torna mais desafiadora devido à presença de dificuldade no relato pela criança e, consequentemente, na identificação de queixas^(5)^. Frequentemente, a tontura pode não ser entendida como um sintoma, podendo ser descrita como um relato de dor, crise histérica ou simplesmente comportamento interpretado como teimosia e aborrecimento^(5)^. Além disso, há limitações nos procedimentos para avaliação nesta população^(6)^. Neste contexto, a posturografia computadorizada tem como intuito facilitar e agilizar a verificação do controle corporal na criança, em diferentes condições sensoriais^(7)^.

Para a realização da avaliação com a posturografia computadorizada, utiliza-se uma plataforma de força que permite verificar as oscilações do centro de gravidade e do centro de pressão (CP), geradas pelo sujeito posicionado sob a plataforma^(8)^. Na análise posturográfica, diversas variáveis podem ser derivadas do estatocinesigrama e do estabilograma, no centro de pressão, durante o exame^(2)^. Essas análises podem ser dividida em dois tipos: análise global, que mensura os padrões de oscilação tanto no domínio do tempo como no domínio das frequências; e análise estrutural, que identifica as subunidades nos dados posturográficos e as relaciona com os processos de controle motor^(2)^.

A plataforma de posturografia computadorizada Horus® apresenta alta precisão para determinar o centro de pressão corporal^(9)^, que é o ponto de aplicação da resultante das forças verticais agindo sobre a superfície de suporte, cujo resultado é dependente da tarefa que é investigada durante a avaliação^(2)^. Destarte, quando o sujeito permanece em pé e estático sobre a plataforma de força durante o teste, são observadas a presença de pequenas oscilações do centro de pressão^(2)^.

Recentemente, pesquisadores propuseram valores normativos para a plataforma computadorizada Horus® em adultos, abrangendo as idades de 40 a 59 anos^(10)^ e de 20 a 89 anos^(11)^, revelando diferenças para as variáveis de idade e sexo. No entanto, ainda não há publicações dos valores normativos na população infantil utilizando este equipamento^(7)^. De maneira geral, encontram-se poucos estudos sobre o uso de posturografia em crianças^(12-14)^, afirmação destacada também anteriormente pela academia Norte Americana de Neurologia^(15)^. Portanto, há uma escassez de publicações com a sugestão de valores normativos para a posturografia do tipo estática, especialmente em crianças pequenas, apesar de ser uma ferramenta frequentemente empregada na avaliação do equilíbrio corporal.

Diante do exposto, o objetivo deste estudo foi propor valores de normatização para a plataforma de posturografia computadorizada Horus® em crianças de 4 a 6 anos, que não apresentassem queixas relacionadas à audição e ao equilíbrio corporal, e com desenvolvimento típico, por meio da análise estatística quantitativa e descritiva, estratificadas por idade e sexo.

## MÉTODO

Trata-se de um estudo transversal, com abordagem quantitativa, aprovado pelo Comitê de Ética de uma Instituição de Ensino Pública, sob número 39835. Além disso, o estudo respeitou as normas e diretrizes regulamentadoras para pesquisa com seres humanos da Resolução 466/12 do Conselho Nacional de Saúde. Foram entregues o Termo de Consentimento Livre e Esclarecido para os responsáveis pelas crianças visando o consentimento da participação na pesquisa, sendo necessária a assinatura do mesmo, e o Termo de Assentimento da Criança para consentimento do participante por meio da assinatura daquelas que já sabiam escrever, bem como, foi solicitado o consentimento do uso de imagem.

A amostra deste estudo consistiu em crianças com idades entre 4 anos e 6 anos e 11 meses, de ambos os sexos, provenientes de creches e escolas públicas municipais e privadas da cidade de Santiago, RS. As crianças não apresentavam queixas relacionadas ao sistema vestibular e/ou auditivo e possuíam desenvolvimento típico. Para recrutar os participantes, foram utilizados métodos de divulgação que incluíram jornais locais, mídias sociais, blogs, rádios e comunicação direta nas escolas.

O tamanho da amostra foi determinado utilizando um método não probabilístico por conveniência, com base em um tamanho de efeito padronizado em 0,9. A estimativa calculada resultou em um tamanho amostral de 192 crianças. O estudo adotou um nível de significância de 0,05 com um poder estatístico de 95% (*Epi Info – StatCalc)*.

Os critérios de inclusão adotados foram: idade entre 4 e 6 anos e 11 meses, apresentar desenvolvimento típico, ausência de queixas otoneurológicas e/ou auditivas. Elencou-se como desenvolvimento típico aquela criança que apresentou evolução conforme esperado para a faixa etária, considerando os marcos do desenvolvimento nos domínios motor grosseiro, motor fino, linguagem e fala, cognitivo e social/emocional, relatados por meio da anamnese realizada com os pais/responsáveis.

Os seguintes critérios de exclusão foram considerados: Crianças com síndromes ou anormalidades craniofaciais; peso corporal inferior a 15kg; apresentar alterações otológicas; queixa de cefaléia; possuir histórico de quedas frequentes e/ou presença de tontura, vertigem, cinetose; possuir dificuldade visual e/ou comprometimento motor, que pudesse comprometer a realização da avaliação; fazer uso de medicamentos com ação sobre o sistema vestibular ou sistema nervoso central; não compreender ou não conseguir, por qualquer razão, realizar os procedimentos e concluir a bateria de avaliação.

A primeira etapa de avaliação consistiu na anamnese realizada com os pais/responsáveis para conhecimento do desenvolvimento infantil até o momento da avaliação, além de verificar possíveis queixas auditivas e/ou relacionadas ao equilíbrio corporal.

Após, verificaram-se as medidas antropométricas como a altura da criança, com uso de fita métrica fixada na parede da sala de avaliação, e o peso, registrado em balança digital, sendo esta medida confirmada posteriormente na realização do exame de equilíbrio pela plataforma Horus®. Para a triagem da acuidade visual, utilizou-se o optotipo direcional “E” de Snellen, seguindo as orientações para utilização da ferramenta conforme o Ministério da Saúde^(16)^.

A avaliação auditiva periférica foi realizada por meio de inspeção visual do meato acústico externo e dos exames de audiometria tonal, utilizando o protocolo de triagem (método de varredura^(17)^ na intensidade de 20dB nas frequências de 1000, 2000 e 4000 Hz, com adição de frequência grave 500 Hz, em ambas as orelhas, avaliadas individualmente (audiometria conduzida com o equipamento *Callisto*, em cabine acústica, utilizando fone do tipo TDH-39), sendo consideradas aptas as crianças que responderam à todas as frequências; Emissões otoacústicas transientes do tipo triagem (equipamento *AccuScreen* por meio de fones de inserção), devendo haver presença de resposta em ambas as orelhas; Timpanometria de sonda 226Hz com pesquisa de reflexos acústicos contralaterais nas frequências de 500, 1000, 2000 e 4000 Hz (equipamento *Zodiac* com fones de inserção e sonda), sendo consideradas respostas adequadas a presença de reflexos e curva timpanométrica do tipo A. Todos os equipamentos estavam calibrados conforme as normas ISO 8253-1.

O último exame realizado foi a posturografia computadorizada, com o equipamento Horus®, da empresa Contronic, adaptado especialmente para esta pesquisa pelo fabricante devido à necessidade de adequação do peso mínimo para a população do estudo. A posturografia Horus® possui uma “Plataforma de Força” que permite determinar o Centro de Pressão (CP) por meio de quatro sensores de força dispostos nos quatro cantos em posição retangular. Esses sensores possibilitam a mensuração dos componentes de força nas direções ânteroposterior, médio-lateral e vertical, atuando sobre a plataforma. Esta é conectada via USB a um *notebook* contendo o *software* Horus® previamente instalado, o qual registra os parâmetros no domínio de tempo e frequência e exibe as respostas no computador, gerando dados quantitativos e objetivos por meio das provas da posturografia. Neste estudo, foi utilizado um *notebook* da marca *Asus,* modelo X450CA.

As imagens necessárias para a realização da avaliação foram apresentadas em televisão de 40 polegadas, da marca *Sony*, a posicionada a uma distância de 1 metro da plataforma e do ponto de posicionamento da criança. A televisão foi posicionada de modo que a tela ficasse no nível da altura dos olhos. Para realizar a avaliação, a criança foi orientada a tirar os sapatos, ficando de pés descalços; subir em uma plataforma de 5 cm de altura; permanecer ereta com os pés afastados e confortáveis durante 30 segundos, estando eles alinhados na linha horizontal da plataforma, afastados de forma simétrica da linha anteroposterior; o dedo hálux apontando entre 0 e 15 graus; ficar com os olhos abertos e olhando para a televisão; olhar as imagens que iriam surgir; e, por fim, ficar com os olhos fechados. Após, foi adicionada uma almofada de espuma com 5cm de altura e D33 de densidade, ficando a 10cm do chão, sendo a criança orientada novamente na nova condição de exame. A avaliação completa foi composta por oito etapas, sendo elas:

1. Teste de Limite de Estabilidade (LE) na plataforma sem almofada, a criança foi instruída a subir na plataforma e inclinar o corpo para frente, voltando ao centro, inclinar para trás, voltando ao centro, inclinar o corpo para a direita, voltando ao centro, e inclinar o corpo para a esquerda, voltando ao centro, repetindo esses movimentos. A movimentação deveria ser realizada apenas pelos tornozelos, sem pressa, sem movimentos dos quadris e/ou ombros, e sem retirar os pés da plataforma. Durante esta etapa, os pontos extremos em cada direção, registrados pela movimentação do corpo, permitiram definir o valor da elipse de estabilidade, que serviu como referência para as etapas subsequentes..
  1. Condição sensorial 1, plataforma sem almofada: permanecer em pé com os olhos abertos, estática, olhando para um ponto amarelo fixo com tamanho de 10% contra um fundo preto, projetado na televisão;
  2. Condição sensorial 2, plataforma sem almofada: permanecer em pé com os olhos fechados, sem se movimentar;
  3. Condição sensorial 3, plataforma com adição da almofada: permanecer em pé com os olhos abertos, estática, olhando para um ponto amarelo fixo com tamanho de 10% contra um fundo preto, projetado na televisão;
  4. Condição sensorial 4, plataforma com adição da almofada: permanecer em pé com os olhos fechados, sem se movimentar;
  5. Condição sensorial 5, plataforma com adição da almofada: permanecer em pé, estática, olhando na televisão o vídeo de barras nas cores preto e branco em efeito optocinético horizontal movimentando-se para a direita, na velocidade de 10%;
  6. Condição sensorial 6, plataforma com adição da almofada: permanecer em pé, estática, olhando na televisão um vídeo contendo barras nas cores preto e branco em efeito optocinético horizontal movimentando-se para a esquerda, na velocidade de 10%;
  7. Condição sensorial 7, plataforma com adição da almofada: permanecer em pé, estática, olhando na televisão um vídeo de um túnel composto por barras finas movimentando-se para frente na velocidade de 4%, sem rotação.

As orientações para as etapas de avaliação acima foram fornecidas em linguagem simples, visando facilitar a compreensão das crianças. Além disso, a examinadora demonstrou para a criança os movimentos para realizar o registro do LE e a posição “parada” nas demais condições de teste, previamente à realização da avaliação. Na Figura 1, pode-se observar o posicionamento do equipamento na sala de avaliação e a posição da criança nas sete condições de teste.

**Figura 1:** Imagem do posicionamento da plataforma Horus® na sala de avaliação e na sequência condições de exame 1, 2, 3, 4, 5, 6, e 7, respectivamente

Para manter as crianças imóveis, foram utilizadas estratégias como o auxílio de boneco posicionado no chão ao lado da plataforma, “em posição estátua”, simulando a realização da avaliação junto à criança; foi sugerido à criança que, ao olhar para a imagem apresentada na tela da televisão, sem se movimentar, poderia aparecer um “pintinho” atrás da bola amarela nas condições 1 e 3, e uma “zebra” nas condições 5, 6 e 7; também foi oferecido auxílio dos familiares/acompanhantes, “brincando de estátua” e realizando as avaliações próximo à criança; além disso, foi realizada a contagem normal ou regressiva em voz alta para cronometrar o tempo de 30 segundos.

A avaliação foi realizada na presença de duas avaliadoras, as quais monitoraram o comportamento da criança durante a avaliação e analisaram separadamente as respostas, visando melhorar a confiabilidade dos achados. Posteriormente, os dados quantificados foram tabulados. Ao identificar qualquer alteração nas avaliações, os pais/responsáveis foram orientados e a criança encaminhada para os devidos tratamentos e avaliações complementares necessários.

Registraram-se os valores das oscilações corporais sob a plataforma durante o tempo de realização em cada condição de teste pelo estabilograma, no sentido de movimentação médio-lateral para a esquerda e para direita, e anteroposterior para frente e para trás, pela medida de amplitude. Na análise pelo estatocinesigrama, tem-se o mapa referente ao deslocamento do CP no eixo médio-lateral e ao deslocamento do CP no eixo anteroposterior, com nuvem de pontos de elipse de confiança de 95%.

Neste estudo, os dados analisados nas respostas obtidas na plataforma foram os seguintes: LE, que corresponde à medida que o corpo consegue movimentar-se em relação ao centro de gravidade sem ter que alterar sua base de suporte, e a área do LE que se refere à região em que o corpo consegue realizar essa ação, em mm^2^; O Elipse de Confiança (EC), caracteriza-se como o registro quantitativo de deslocamento ou dificuldade do sujeito em manter-se sem oscilação em relação ao CP, em mm^2^; a velocidade, que refere-se à velocidade média em relação ao CP, quanto menor seu valor, em mm/s, melhor será o equilíbrio corporal; e o Equilíbrio Funcional Residual (EFR), que demonstra a área ainda disponível com segurança para a oscilação do sujeito, registrado em porcentagem pela relação entre a área do LE e a área do EC.

Os dados foram analisados no *software Statistical Package for Social Sciences (SPSS)* para *Windows,* versão 22.0. Os dados categóricos foram apresentados em frequência relativa, e os dados quantitativos pela média e desvio padrão. Foram utilizados os testes estatísticos post hoc Dunn-Bonferroni para comparações par-a-par nas idades, Teste U de Mann-Whitney para análise entre os sexos e o teste estatístico não paramétrico Kruskal-Wallis. Foram considerados significativos os valores de p<0,05.

## RESULTADOS

Compareceram para avaliação 231 crianças, sendo excluídas 15 por não estarem dentro dos critérios de inclusão. Ao final, participaram deste estudo 216 crianças, sendo 49,5% do sexo feminino (107 meninas) e 50,5% do sexo masculino (109 meninos). Para melhor verificação dos dados e análise de possíveis associações, os sujeitos foram divididos em três grupos conforme a idade: 4 anos a 4 anos e 11 meses (n= 77), 5 anos a 5 anos e 11 meses (n=69), 6 anos a 6 anos e 11 meses (n=70).

As tabelas apresentadas na sequência são extensas; entretanto, acredita-se que é imprescindível expor os valores detalhadamente, em consonância com o objetivo deste estudo, que é propor valores de normatização da plataforma de posturografia computadorizada Horus®. Cabe ressaltar que estes valores ainda não foram descritos e propostos para a faixa etária pesquisada, neste equipamento específico. Dessa forma, espera-se que esses achados possam auxiliar outros pesquisadores em futuros estudos desenvolvidos nessa população.

Os valores referentes ao limite de estabilidade, segundo as variáveis idade e sexo, podem ser observados na Tabela 1.

**Tabela 1:** Análise do limite de estabilidade (mm^2^) segundo as variáveis idade e sexo, para a plataforma Horus® (n= 216)

GRUPO	N	Média	Desvio Padrão	Mínimo	Percentil 05	Percentil 25	Mediana	Percentil 75	Percentil 95	Máximo	p-valor*
COMPARAÇÃO ENTRE IDADES											
4 ANOS (A)	77	8689	4048,4	1959,3	3362,5	5615,2	7745,6	11149,7	16891,2	18741,9	<0,001**
5 ANOS (B)	69	10646,4	3613,7	3168,4	5511,4	8444,3	10171,5	12409,6	16732,4	21518,2	A≠(B,C)
6 ANOS (C)	70	11342,9	4142,2	3837,6	5318,2	8064,1	11313,9	14533,7	17371,3	21182,8	
COMPARAÇÃO POR SEXO – 4 ANOS											
FEMININO	38	7540	3793,6	3115,6	3362,5	5071,3	6550,8	8623,5	17409,7	17776	0,007**
MASCULINO	39	9808,7	4019,9	1959,3	3136,4	6616	9567,1	12481,4	16785,2	18741,9	
COMPARAÇÃO POR SEXO – 5 ANOS											
FEMININO	35	10231,6	4061,1	3168,4	3176,3	8026,5	9601,4	11997,2	21368,6	21518,2	0,275
MASCULINO	34	11073,3	3089,7	5511,4	7398,2	8677,1	10299,2	13883,2	15914,1	18196,6	
COMPARAÇÃO POR SEXO – 6 ANOS											
FEMININO	34	10465,2	4211,5	3837,6	3931	7166,2	10298,4	13492,9	17371,3	21182,8	0,065
MASCULINO	36	12171,9	3955,6	5437,9	5837,2	8811,1	12740,1	15540	18086	18109,5	

Legenda: N = voluntários

* Valor-P referente ao Teste de Kruskal-Wallis para análise entre idades e Teste U de Mann-Whitney para análise entre sexo;

** Resultado estatisticamente significativo

A Tabela 2 expõe os achados obtidos na avaliação do EFR, com as respostas são apresentadas nas sete condições de exame, por faixa etária.

**Tabela 2:** Análise dos valores numéricos do equilíbrio funcional residual, nas sete condições do exame, para a plataforma Horus® por idade (n= 216)

IDADE	N	VARIÁVEL	Média ± DP	Mínimo	Percentil 05	Percentil 25	Mediana	Percentil 75	Percentil 95	Máx.	p-valor*
4 ANOS (A)	77	EC, C1	778,0 ± 510,8	80,6	179,7	419,5	662,1	996,5	1793,4	2610,2	<0,001** A≠C, B≠C
5 ANOS (B)	69	EC, C1	689,9 ± 755,1	82,9	151,6	323	543,8	822,8	1327,6	5731	
6 ANOS (C)	70	EC, C1	469,6 ± 366,6	69,3	126,7	227,1	393,2	599,4	1036,1	2169,1	
4 ANOS (A)	77	VelML, C1	10,1 ± 4,5	3,5	4,2	6,5	8,4	12,8	19,3	21,7	0,009** A≠C
5 ANOS (B)	69	VelML, C1	8,5 ± 3,5	2,9	4,1	6,3	8	10,2	16,6	19,5	
6 ANOS (C)	70	VelML, C1	7,8 ± 3,4	2,9	3,6	5,1	7,2	9,6	14,2	17,7	
4 ANOS (A)	77	VelAP, C1	15,1± 4,7	6,8	8,8	11,5	14	18,7	24,3	29,1	<0,001** A≠(B,C)
5 ANOS (B)	69	VelAP, C1	12,4 ± 3,9	6,2	7	10	11,9	14,1	20,7	25,2	
6 ANOS (C)	70	VelAP, C1	10,8 ± 3,5	5,4	5,8	7,9	11	13,1	17	21,2	
4 ANOS (A)	77	EFR, C1	90,2 ± 6,1	71,5	76,8	87,6	91	95	97,4	99	<0,001** A≠B, A≠C, B≠C
5 ANOS (B)	69	EFR, C1	93,8 ± 4,1	83,6	85,2	91,6	94,8	97	98,7	99,1	
6 ANOS (C)	70	EFR, C1	95,8 ± 2,8	84,7	90,8	94,4	96,4	97,7	98,9	99,3	
4 ANOS (A)	77	EC, C2	955,9 ± 637,3	100,5	223	454,9	811,6	1240	1957,5	3301,8	<0,001** A≠(B,C)
5 ANOS (B)	69	EC, C2	687,0 ± 536,1	68,7	172,4	375,9	560,6	783,8	1448	3959,8	
6 ANOS (C)	70	EC, C2	543,2 ± 328,5	88,4	139,5	264,4	511,3	750,3	1160,4	1725,5	
4 ANOS (A)	77	VelML, C2	12,5 ± 5,4	3,5	5,7	8,6	11,8	15,9	25,2	29,7	0,001** A≠C
5 ANOS (B)	69	VelML, C2	10,4 ± 4,1	3,5	4,3	7,8	9,8	12,6	16,8	25,6	
6 ANOS (C)	70	VelML, C2	9,5 ± 4,1	3,4	3,7	6,2	9,4	11,5	18,1	22,2	
4 ANOS (A)	77	VelAP, C2	21,1 ± 6,3	9,8	11,5	15,9	20,1	25	32,5	35,5	<0,001** A≠(B,C)
5 ANOS (B)	69	VelAP, C2	17,6 ± 5,5	8	9,6	13,7	16,8	21	27,2	36,1	
6 ANOS (C)	70	VelAP, C2	16,0 ± 5,8	7,2	7,9	12,5	15,6	18,8	27,7	37,3	
4 ANOS (A)	77	EFR, C2	88,4 ± 6,4	74,1	75,7	83,9	90,4	93,1	97,2	98,7	<0,001** A≠(B,C)
5 ANOS (B)	69	EFR, C2	93,1 ± 4,7	74,7	84,5	90,8	94,5	96,4	98,5	99,1	
6 ANOS (C)	70	EFR, C2	95,0 ± 3,0	81,7	90,2	93,6	95,3	97,4	98,5	99,5	
4 ANOS (A)	77	EC, C3	1036,4 ± 589,6	204,1	266,8	648,9	944,2	1385,3	2263	3219,4	<0,001** A≠(B,C)
5 ANOS (B)	69	EC, C3	798,0 ± 427,7	214,9	294,4	515,3	699	1015,5	1583,2	2612,8	
6 ANOS (C)	70	EC, C3	679,6 ± 325,5	160,6	205,9	439,1	627,6	854,4	1367,8	1562,3	
4 ANOS (A)	77	VelML, C3	14,4 ± 4,9	5,3	6,5	11	13,3	18,8	23	25,4	0,004** A≠C
5 ANOS (B)	69	VelML, C3	12,6 ± 4,1	6	7,3	9,8	12,1	15	20	27,2	
6 ANOS (C)	70	VelML, C3	11,9 ± 4,2	5,5	6,5	8,6	11,1	14	19,9	22,9	
4 ANOS (A)	77	VelAP, C3	19,43 ± 5,26	7,5	11,2	16,1	18,6	22,3	29,4	34,7	<0,001** A≠(B,C)
5 ANOS (B)	69	VelAP, C3	16,84 ± 4,95	9,6	10,7	12,8	15,4	20	25,7	31,1	
6 ANOS (C)	70	VelAP, C3	15,70 ± 5,03	8,6	9,5	12,3	15,1	17,5	24,8	38,7	
4 ANOS (A)	77	EFR, C3	86,9 ± 7,3	49,9	73,7	83,2	88,4	91,6	96,2	98,2	<0,001** A≠(B,C)
5 ANOS (B)	69	EFR, C3	91,9 ± 4,2	74,5	84,9	89,8	92,4	95,1	97,2	97,8	
6 ANOS (C)	70	EFR, C3	93,5 ± 3,2	80,4	87,8	92,1	93,9	95,4	97,8	98,4	
4 ANOS (A)	77	EC, C4	1530,9±661,5	438	639,2	998,7	1522,1	1979,2	2815	3500,9	0,007** A≠(B,C)
5 ANOS (B)	69	EC, C4	1317,6±718,2	457,7	529,1	898,8	1134,2	1541,8	2770	4693,8	
6 ANOS (C)	70	EC, C4	1220,7±574,1	369,5	468	765,4	1227,4	1523,4	2432,1	3016,9	
4 ANOS (A)	77	VelML, C4	21,1 ± 7,4	8,5	11,7	15,8	19,6	24,4	38,9	43,6	0,404
5 ANOS (B)	69	VelML, C4	19,6 ± 6,9	8	9,3	15,3	18,2	23,1	32,6	37,7	
6 ANOS (C)	70	VelML, C4	19,8 ± 7,1	8,6	10,6	13,6	18,9	24	32,4	44,9	
4 ANOS (A)	77	VelAP, C4	31,3 ± 7,9	15,4	18,1	26,3	30,4	36,6	44,1	52,8	<0,001** A≠(B,C)
5 ANOS (B)	69	VelAP, C4	27,4 ± 8,6	13,8	14,8	21,1	26,4	31,4	42,8	52,2	
6 ANOS (C)	70	VelAP, C4	26,3 ± 8,9	13,4	15	19,6	24,5	30,5	48,7	51,2	
4 ANOS (A)	77	EFR, C4	80,6 ± 8,1	52,4	66,6	75,5	82	86	91,2	95,7	<0,001** A≠(B,C)
5 ANOS (B)	69	EFR, C4	86,6 ± 7,9	49,1	73,9	81,9	88,1	91,9	95,4	96,5	
6 ANOS (C)	70	EFR, C4	88,6 ± 4,9	72,2	81,4	85,2	89	91,3	96,4	97	
4 ANOS (A)	77	EC, C5	1137,0±531,6	385	452,7	714,2	1059,8	1425,3	2029,4	2810,9	0,028** A≠C
5 ANOS (B)	69	EC, C5	1039,0±614,0	371	423,4	679,2	913,7	1220,1	2088,6	4294	
6 ANOS (C)	70	EC, C5	896,9 ± 399,2	207,1	379,3	600,3	872,9	1154,9	1615,2	2311,6	
4 ANOS (A)	77	VelML, C5	15,9 ± 5,0	6,5	8,7	12,1	15,2	19,1	24,1	35	0,727 A≠C
5 ANOS (B)	69	VelML, C5	15,4 ± 4,8	7	9,4	12,6	14,5	17,4	25,7	33,7	
6 ANOS (C)	70	VelML, C5	15,5 ± 5,5	6,5	7,7	10,8	15	18,6	24,9	29,9	
4 ANOS (A)	77	VelAP, C5	21,3 ± 4,9	11,6	13,5	17,5	21,4	25,3	30,4	32,6	0,14
5 ANOS (B)	69	VelAP, C5	20,4 ± 5,1	11,5	12,8	17,3	19,5	23,4	30,7	34,9	
6 ANOS (C)	70	VelAP, C5	19,8 ± 6,0	10,4	11,4	15,6	19,4	23,7	30,3	43,3	
4 ANOS (A)	77	EFR, C5	85,7 ± 6,0	66,1	74,6	82,3	86	90,1	93,6	95,7	<0,001** A≠(B,C)
5 ANOS (B)	69	EFR, C5	89,7 ± 5,3	72,6	80,3	87,6	90,6	93,9	95,9	96,1	
6 ANOS (C)	70	EFR, C5	91,5 ± 3,5	82,5	85,3	89,2	92	94,1	96,1	98	
4 ANOS (A)	77	EC, C6	1199,9±639,6	347,8	518,9	714,9	1148,6	1502,2	2603,1	3715,5	0,049**
5 ANOS (B)	69	EC, C6	1012,4±612,7	270,8	410,9	692	899,7	1270,1	1938,1	4195,4	
6 ANOS (C)	70	EC, C6	6936,7±392,3	173,5	291,5	661,3	949,2	1198,7	1545,1	2056,3	
4 ANOS (A)	77	VelML, C6	16,3 ± 5,4	8	8,4	12,4	15,4	19,4	27,4	30,4	0,666
5 ANOS (B)	69	VelML, C6	15,7 ± 5,6	7,3	8,7	12	14,6	18,5	24,7	35,2	
6 ANOS (C)	70	VelML, C6	15,5 ± 5,4	5,3	8,8	12	14,7	18,8	24,2	32,3	
4 ANOS (A)	77	VelAP, C6	21,7 ± 5,8	13,4	14,5	17,7	19,8	25,5	33,2	38,4	0,028** A≠C
5 ANOS (B)	69	VelAP, C6	21,0 ± 10,1	10,9	12,6	16,2	19,1	22,4	32,5	92,3	
6 ANOS (C)	70	VelAP, C6	19,2 ± 5,5	10,1	12,7	15,1	18,5	21,7	30,2	36,4	
4 ANOS (A)	77	EFR, C6	84,9 ± 7,0	55,9	73,1	81,9	86	89,8	94,2	96,5	<0,001** A≠(B,C)
5 ANOS (B)	69	EFR, C6	89,8 ± 5,6	72,5	80,3	86,6	91,2	94,1	96,2	97,3	
6 ANOS (C)	70	EFR, C6	91,0 ± 4,0	79,5	83,4	88,3	91,7	94	97,2	98	
4 ANOS (A)	77	EC, C7	1098,8±478,7	355,6	480,6	741,4	1073	1371,5	2004,9	2513,1	0,001** A≠C, B≠C
5 ANOS (B)	69	EC, C7	1016,0±535,2	343,4	423,6	690,2	905,6	1188	1969	3747,5	
6 ANOS (C)	70	EC, C7	811,0 ± 383,6	271,7	304,5	562,4	747	1030,9	1428	1973,8	
4 ANOS (A)	77	VelML, C7	13,9 ± 3,6	6,7	8,3	11,4	13,5	16,2	21,2	22,1	0,115
5 ANOS (B)	69	VelML, C7	13,3 ± 3,8	6	8,2	10,7	12,8	15,4	19,4	24,7	
6 ANOS (C)	70	VelML, C7	12,7 ± 3,8	4,9	7,8	9,9	12,3	15,3	18,5	24,2	
4 ANOS (A)	77	VelAP, C7	21,5 ± 4,5	12,4	14,5	18,5	21,6	24,8	30	31,2	<0,001** A≠C
5 ANOS (B)	69	VelAP, C7	20,0 ± 4,7	10,4	13,4	17	18,9	22,8	28,3	31,1	
6 ANOS (C)	70	VelAP, C7	18,4 ± 5,3	10,1	11,3	14,6	17,7	20,6	27	39,6	
4 ANOS (A)	77	EFR, C7	85,7 ± 6,9	61,7	73,3	82,6	87,3	91,1	93,6	94,6	<0,001** A≠(B,C)
5 ANOS (B)	69	EFR, C7	89,7 ± 58,9	67,9	76,1	87,4	91,1	93,9	95,9	96,7	
6 ANOS (C)	70	EFR, C7	91,9 ± 4,4	80,3	82,8	90,1	92,9	95,2	97,5	98,1	

Legenda: N = voluntários; DP = desvio padrão; EC = elipse de confiança; VelML = velocidade média-lateral; VelAP = velocidade média anteroposterior; EFR = equilíbrio funcional residual; C1 = condição olhos abertos superfície estável (mm^2^); C2 = condição olhos fechados superfície estável (mm^2^); C3 = condição olhos abertos superfície instável (mm^2^); C4 = condição olhos fechados superfície instável (mm^2^); C5 = condição estimulação optocinética para a direita em superfície instável (mm^2^); C6 = condição estimulação optocinética para a esquerda em superfície instável (mm^2^); C7 = condição estimulação túnel em superfície instável (mm^2^)

* Valor-P referente ao teste de Kruskal-Wallis para comparação dos valores entre 3 grupos e teste post hoc Dunn-Bonferroni para comparação par-a-par nas idades;

** Resultado estatisticamente significante

A Tabela 3 apresenta os achados que mostraram diferença estatisticamente significantes na avaliação do equilíbrio funcional residual, por idade e sexo, nas sete condições do exame.

**Tabela 3:** Resultados estatisticamente significantes obtidos na pesquisa do Equilíbrio funcional residual, por idade, nas sete condições do exame, para a plataforma Horus® na comparação pela variável sexo

IDADE E SEXO	N	VARIÁVEL	Média ± DP	Mínimo	Percentil 05	Percentil 25	Mediana	Percentil 75	Percentil 95	Máximo	p-valor*
4 ANOS, F	38	EC, C1	617,3 ± 412,5	80,6	131,0	309,0	549,8	738,0	1715,2	1780,7	0,004
4 ANOS, M	39	EC, C1	934,6 ± 552,5	118,4	240,5	537,8	814,3	1299,4	1913,4	2610,2	
5 ANOS, F	35	EC, C1	498,1 ± 320,1	82,9	105,8	192,9	444,8	685,3	1062,2	1295,3	0,019
5 ANOS, M	34	EC, C1	887,2 ± 995,2	181,5	204,6	448,9	631,5	887,2	2428,1	5731,0	
4 ANOS, F	38	VelML, C1	8,7 ± 3,6	3,5	4,0	6,0	7,5	12,4	15,0	15,4	0,012
4 ANOS, M	39	VelML, C1	11,4 ± 4,9	4,2	4,5	7,4	11,3	14,7	20,2	21,7	
5 ANOS, F	35	VelML, C1	7,5 ± 2,5	2,9	3,1	5,8	7,2	9,0	12,5	13,0	0,05
5 ANOS, M	34	VelML, C1	9,6 ± 4,1	4,1	4,3	7,0	8,5	10,8	19,4	19,5	
6 ANOS, F	34	VelML, C1	7,0 ± 3,3	3,3	3,6	4,7	6,1	8,3	14,4	17,5	0,019
6 ANOS, M	36	VelML, C1	8,5 ± 3,2	2,9	3,2	6,7	8,3	10,5	14,2	17,7	
4 ANOS, F	38	VelAP, C1	13,9 ± 4,5	6,8	8,5	10,0	12,9	17,4	22,3	24,9	0,016
4 ANOS, M	39	VelAP, C1	16,4 ± 4,6	9,6	10,2	12,9	15,7	19,5	24,8	29,1	
5 ANOS, F	35	VelAP, C1	11,3 ± 3,4	6,2	6,3	9,3	10,4	13,4	17,1	20,7	0,007
5 ANOS, M	34	VelAP, C1	13,6 ± 4,0	7,0	7,2	11,3	13,3	15,3	21,5	25,2	
6 ANOS, F	34	VelAP, C1	9,8 ± 3,3	5,4	5,5	7,6	9,1	11,9	14,6	21,2	0,011
6 ANOS, M	36	VelAP, C1	11,8 ± 3,3	6,0	6,1	9,7	11,9	13,7	17,5	19,6	
4 ANOS, F	38	EC, C2	787,2 ± 588,1	100,5	168,0	412,2	602,3	1036,1	1889,2	2784,4	0,007
4 ANOS, M	39	EC, C2	1120,4 ± 647,5	223,0	248,1	659,4	1045,3	1575,0	2319,4	3301,8	
6 ANOS, F	34	EC, C2	442,0 ± 264,3	88,4	127,8	220,8	364,4	619,7	940,0	1175,8	0,017
6 ANOS, M	36	EC, C2	638,7 ± 357,2	138,1	139,5	326,1	656,1	872,9	1225,5	1725,5	
5 ANOS, F	35	VelML, C2	9,1 ± 3,0	3,5	3,8	7,2	9,2	11,5	14,3	14,7	0,027
5 ANOS, M	34	VelML, C2	11,6 ± 4,6	5,1	6,2	8,1	11,4	13,9	22,5	25,6	
6 ANOS, F	34	VelML, C2	8,2 ± 3,8	3,4	3,5	5,8	7,4	9,8	16,3	20,4	0,004
6 ANOS, M	36	VelML, C2	10,7 ± 4,1	3,4	3,7	8,5	10,5	12,6	19,2	22,2	
4 ANOS, F	38	VelAP, C2	19,6 ± 6,8	9,8	10,0	14,2	18,1	23,3	34,2	35,5	0,021
4 ANOS, M	39	VelAP, C2	22,5 ± 5,5	13,7	14,4	18,2	22,4	25,5	32,5	33,2	
5 ANOS, F	35	VelAP, C2	16,2 ± 5,0	8,0	8,9	12,2	16,1	19,1	25,1	29,2	0,044
5 ANOS, M	34	VelAP, C2	19,0 ± 5,6	12,6	13,3	14,6	17,8	22,4	32,7	36,1	
6 ANOS, F	34	VelAP, C2	13,6 ± 4,2	7,2	7,4	10,8	13,3	16,1	22,2	22,9	>0,001
6 ANOS, M	36	VelAP, C2	18,2 ± 6,2	9,0	9,1	15,1	16,9	20,6	33,9	37,3	
4 ANOS, F	38	EC, C3	844,7 ± 455,7	204,1	233,8	466,6	762,9	1076,4	1635,5	1855,6	0,005
4 ANOS, M	39	EC, C3	1223,1±648,4	233,2	299,0	823,5	1133,4	1504,6	3079,4	3219,4	
5 ANOS, F	35	EC, C3	637,8 ± 261,8	214,9	252,6	445,2	612,4	800,8	1080,9	1139,3	0,001
5 ANOS, M	34	EC, C3	-5,5 ± 9,2	-25,5	-22,7	-9,3	-5,7	,9	9,1	9,9	
4 ANOS, F	38	VelML, C3	12,8 ± 5,1	5,3	5,6	10,1	12,3	14,1	23,1	25,4	0,003
4 ANOS, M	39	VelML, C3	15,9 ± 4,3	8,5	8,6	12,0	14,9	19,1	22,7	24,5	
5 ANOS, F	35	VelML, C3	11,3 ± 2,9	6,0	7,3	9,4	11,2	13,8	15,7	18,5	0,015
5 ANOS, M	34	VelML, C3	13,9 ± 4,8	6,0	6,6	10,4	13,1	15,9	23,8	27,2	
6 ANOS, F	34	VelML, C3	10,6 ± 4,0	5,5	6,1	8,0	9,4	12,2	19,1	19,9	0,003
6 ANOS, M	36	VelML, C3	13,2 ± 4,1	5,5	7,4	10,8	12,7	15,8	21,6	22,9	
4 ANOS, F	38	VelAP, C3	18,19 ± 5,51	7,50	9,90	15,00	17,05	21,00	30,50	31,70	0,017
4 ANOS, M	39	VelAP, C3	20,64 ± 4,77	12,00	12,60	17,60	20,90	23,20	28,80	34,70	
5 ANOS, F	35	VelAP, C3	15,39 ± 4,31	9,60	9,70	12,80	13,90	16,30	24,90	27,30	0,009
5 ANOS, M	34	VelAP, C3	18,35 ± 5,18	10,60	10,70	15,00	17,60	20,90	31,00	31,10	
6 ANOS, F	34	VelAP, C3	14,53 ± 5,55	9,40	9,50	11,10	13,40	16,30	26,20	38,70	0,004
6 ANOS, M	36	VelAP, C3	16,80 ± 4,27	8,60	8,80	13,95	16,75	19,00	24,80	26,30	
5 ANOS, F	35	EFR, C3	93,1 ± 3,2	84,8	87,0	90,2	93,8	95,5	97,6	97,8	0,027
5 ANOS, M	34	EFR, C3	90,7 ± 4,8	74,5	79,9	88,3	91,4	94,2	97,0	97,2	
4 ANOS, F	38	EC, C4	1308,8 ± 636,7	438,0	469,7	863,5	1058,6	1712,4	2815,0	3195,3	0,002
4 ANOS, M	39	EC, C4	1747,3 ± 618,9	721,3	729,3	1319,6	1757,6	2124,3	3019,3	3500,9	
6 ANOS, F	34	EC, C4	1018,9 ± 515,1	369,5	438,0	608,4	942,0	1279,0	2030,3	2612,3	0,001
6 ANOS, M	36	EC, C4	1411,3 ± 568,0	468,0	524,6	1091,8	1343,1	1662,8	2847,2	3016,9	
6 ANOS, F	34	VelML, C4	16,7 ± 5,5	8,6	9,9	12,4	16,1	19,9	28,0	29,6	<0,001
6 ANOS, M	36	VelML, C4	22,9 ± 7,2	12,5	12,5	18,6	22,0	26,1	33,6	44,9	
4 ANOS, F	38	VelAP, C4	29,3 ± 7,1	15,4	16,0	25,3	27,7	31,9	43,5	46,7	0,039
4 ANOS, M	39	VelAP, C4	33,2 ± 8,3	17,8	20,0	26,8	32,2	40,6	46,3	52,8	
5 ANOS, F	35	VelAP, C4	25,4 ± 8,7	13,8	13,9	18,9	25,1	28,9	44,1	47,7	0,028
5 ANOS, M	34	VelAP, C4	29,4 ± 8,0	15,5	18,2	22,7	29,7	34,8	42,3	52,2	
6 ANOS, F	34	VelAP, C4	22,4 ± 5,3	13,4	14,1	18,5	21,7	25,5	33,4	34,0	0,001
6 ANOS, M	36	VelAP, C4	30,0 ± 10,0	14,9	16,4	23,3	28,7	34,0	49,2	51,2	
4 ANOS, F	38	EC, C5	919,2 ± 421,2	385,0	395,3	594,7	795,7	1167,0	1840,0	1914,1	<0,001
4 ANOS, M	39	EC, C5	1349,2 ± 546,6	540,5	572,9	905,6	1320,8	1800,9	2462,0	2810,9	
5 ANOS, F	35	EC, C5	876,5 ± 371,6	389,9	415,6	545,0	786,3	1143,9	1606,9	1620,0	0,033
5 ANOS, M	34	EC, C5	1206,2 ± 760,0	371,0	478,1	795,8	1056,7	1264,2	2758,2	4294,0	
6 ANOS, F	34	EC, C5	735,6 ± 289,1	207,1	250,3	523,1	705,7	949,3	1247,7	1289,9	0,002
6 ANOS, M	36	EC, C5	1049,2 ± 431,5	377,3	389,1	787,6	962,2	1343,5	1808,2	2311,6	
4 ANOS, F	38	VelML, C5	14,4 ± 4,7	6,5	7,8	11,3	13,1	18,4	23,7	26,1	0,01
4 ANOS, M	39	VelML, C5	17,3 ± 4,9	9,9	10,1	12,8	17,5	19,6	24,2	35,0	
6 ANOS, F	34	VelML, C5	13,4 ± 4,2	6,5	7,4	10,0	12,9	17,0	19,8	24,6	0,005
6 ANOS, M	36	VelML, C5	17,4 ± 5,9	6,8	8,1	12,2	17,3	21,3	28,3	29,9	
4 ANOS, F	38	VelAP, C5	20,1 ± 4,8	11,6	12,6	16,6	19,1	22,4	30,4	31,1	0,033
4 ANOS, M	39	VelAP, C5	22,4 ± 4,7	13,5	13,8	18,5	22,6	26,0	30,7	32,6	
6 ANOS, F	34	VelAP, C5	18,2 ± 4,4	10,4	11,6	15,3	18,2	21,1	26,1	30,3	0,04
6 ANOS, M	36	VelAP, C5	21,4 ± 6,8	11,3	11,3	17,6	20,0	25,0	35,6	43,3	
4 ANOS, F	38	EC, C6	955,1 ± 443,5	427,4	452,1	602,3	842,0	1185,4	2091,9	2385,1	0,001
4 ANOS, M	39	EC, C6	1438,4 ± 713,0	347,8	537,6	884,6	1223,4	1735,8	2857,8	3715,5	
4 ANOS, F	38	VelML, C6	15,1 ± 5,4	8,0	8,3	10,9	14,0	18,4	27,4	30,4	0,034
4 ANOS, M	39	VelML, C6	17,4 ± 5,1	9,7	10,6	13,7	16,0	20,9	29,6	29,9	
6 ANOS, F	34	VelML, C6	14,2 ± 5,0	5,3	7,1	10,6	14,2	15,8	27,4	30,0	0,042
6 ANOS, M	36	VelML, C6	16,7 ± 5,5	7,4	9,0	12,8	16,0	20,8	24,2	32,3	
4 ANOS, F	38	VelAP, C6	19,9 ± 4,5	13,4	13,5	16,3	18,8	23,6	28,6	28,7	0,015
4 ANOS, M	39	VelAP, C6	23,4 ± 6,5	13,6	14,5	18,2	22,2	27,4	37,8	38,4	
6 ANOS, F	34	VelAP, C6	17,9 ± 5,3	10,1	12,7	14,1	16,6	19,4	29,5	34,4	0,013
6 ANOS, M	36	VelAP, C6	20,5 ± 5,4	10,2	12,6	17,1	20,7	23,1	30,3	36,4	
4 ANOS, F	38	EC, C7	987,3 ± 455,2	355,6	399,7	547,9	906,2	1307,6	1972,0	2004,9	0,036
4 ANOS, M	39	EC, C7	1207,5 ± 481,6	493,1	520,7	817,3	1153,2	1451,6	2164,5	2513,1	
6 ANOS, F	34	EC, C7	691,9 ± 316,1	271,7	280,1	479,6	622,1	836,5	1370,0	1410,7	0,012
6 ANOS, M	36	EC, C7	923,5 ± 411,2	381,4	387,3	604,1	825,9	1197,0	1842,4	1973,8	
4 ANOS, F	38	VelML, C7	12,8 ± 3,7	6,7	6,7	9,8	13,0	15,0	21,2	21,4	0,026
4 ANOS, M	39	VelML, C7	14,9 ± 3,3	9,6	10,0	12,0	14,1	17,9	21,2	22,1	
6 ANOS, F	34	VelML, C7	11,1 ± 2,9	4,9	7,0	9,2	10,2	13,0	16,5	17,9	0,001
6 ANOS, M	36	VelML, C7	14,1 ± 4,0	5,1	8,6	11,0	13,7	16,8	21,6	24,2	
6 ANOS, F	34	VelAP, C7	16,6 ± 3,9	10,1	10,8	14,1	16,1	19,6	24,5	25,6	0,009
6 ANOS, M	36	VelAP, C7	20,0 ± 6,0	10,4	12,7	16,0	19,5	20,9	34,4	39,6	

Legenda: N = voluntários; DP = desvio padrão; EC = elipse de confiança; F = feminino; M = masculino; VelML = velocidade média-lateral; VelAP = velocidade média anteroposterior; EFR = equilíbrio funcional residual; C1 = condição olhos abertos superfície estável (mm^2^); C2 = condição olhos fechados superfície estável (mm^2^); C3 = condição olhos abertos superfície instável (mm^2^); C4 = condição olhos fechados superfície instável (mm^2^); C5 = condição estimulação optocinética para a direita em superfície instável (mm^2^); C6 = condição estimulação optocinética para a esquerda em superfície instável (mm^2^); C7 = condição estimulação túnel em superfície instável (mm^2^)

* Valor-P referente ao teste U de Mann-Whitney

Por fim, a análise das respostas nos sistemas sensoriais por faixa etária é apresentada na Tabela 4.

**Tabela 4:** Análise sensorial na comparação por faixa etária, em mm^2^, para a plataforma Horus® (n= 216)

IDADE	SISTEMA	N	Média	Desvio Padrão	Mínimo	Percentil 05	Percentil 25	Mediana	Percentil 75	Percentil 95	Máximo	p-valor*
4 ANOS (A)	SOM	77	98,1	5,1	82,4	86,7	95,7	97,7	100,4	106,3	109,8	0,184
5 ANOS (B)	SOM	69	99,1	3,5	87,7	94,3	97,3	99,1	101,1	103,8	111,8	
6 ANOS (C)	SOM	70	99,2	2,8	92,5	94,8	97,8	99,1	100,3	102,6	111,8	
4 ANOS (A)	VIS	77	96,5	5,5	68,6	88,5	94,0	96,3	99,4	104,8	107,2	0,089
5 ANOS (B)	VIS	69	98,1	3,8	85,1	92,3	96,2	98,2	100,0	103,4	113,0	
6 ANOS (C)	VIS	70	97,6	2,7	88,5	93,3	96,2	98,2	99,1	101,3	105,3	
4 ANOS (A)	VEST	77	89,3	7,6	71,6	76,7	84,6	88,8	95,2	101,5	106,2	0,006** A≠(B,C)
5 ANOS (B)	VEST	69	92,2	7,3	54,6	83,3	89,0	92,9	96,6	100,8	109,6	
6 ANOS (C)	VEST	70	92,5	4,8	74,8	85,2	89,9	93,1	95,3	98,1	110,2	
4 ANOS (A)	DPVD	77	105,8	13,7	11,8	94,9	100,1	106,4	112,0	125,0	127,2	0,024** A≠B
5 ANOS (B)	DPVD	69	104,1	7,8	93,8	95,9	99,6	102,6	106,9	116,1	151,7	
6 ANOS (C)	DPVD	70	103,5	4,1	95,8	97,9	100,5	103,4	106,3	111,1	114,3	
4 ANOS (A)	DPVE	77	105,6	9,4	60,4	93,9	99,6	105,7	110,9	121,2	124,6	0,033** A≠C
5 ANOS (B)	DPVE	69	104,2	7,9	95,1	96,0	100,3	102,4	106,5	115,0	153,0	
6 ANOS (C)	DPVE	70	101,6	11,9	11,5	96,0	99,9	102,5	104,7	110,9	121,9	
4 ANOS (A)	DPVT	77	107,4	7,5	96,1	97,3	102,0	105,8	111,8	122,4	131,5	0,002** A≠(B,C)
5 ANOS (B)	DPVT	69	102,6	12,9	11,8	96,0	99,9	101,7	106,6	113,7	138,3	
6 ANOS (C)	DPVT	70	102,7	12,3	11,5	95,6	100,9	104,0	107,3	111,4	124,0	
4 ANOS (A)	IEC	77	85,4	5,9	59,2	73,0	82,1	87,0	88,9	92,4	96,3	<0,001** A≠(B,C)
5 ANOS (B)	IEC	69	90,0	4,9	73,6	80,3	88,0	90,8	93,8	95,8	96,9	
6 ANOS (C)	IEC	70	91,8	3,1	83,1	86,2	89,9	92,2	93,6	97,0	97,5	

Legenda: N = voluntários; SOM = somatossensorial (%); VIS = visual (%); VEST = vestibular (%); DPVD = dependência visual optocinético para direita (%); DPVE = dependência visual optocinético para esquerda (%); DPVT = dependência visual túnel (%); IEC = índice de equilíbrio composto (%)

* Valor-P referente ao teste de Kruskal-Wallis;

** Resultado estatisticamente significativo

Outros achados importantes referem-se à análise das respostas sensoriais por sexo nas idades de 4 e 5 anos, onde não se verificou diferença estatisticamente significante entre meninos e meninas. Ao passo que, nas crianças com 6 anos, observou-se diferença apenas para as etapas relacionadas ao sistema visual. Foram encontrados achados significantes na dependência para esquerda (p=0,009), onde os meninos apresentaram valores superiores tanto para média, mediana e valor mínimo (percentis 05, 25, 75, e 95), apenas para situação de dependência à esquerda, e na situação de túnel (p=0,04) com valores inferiores em relação às meninas para valor máximo e percentil 95.

## DISCUSSÃO

A plataforma de posturografia estática com provas dinâmicas Horus® e seu *software* foram desenvolvidos por uma empresa brasileira com o objetivo de atender à demanda dos profissionais da saúde. A empresa propõe um equipamento portátil e de fácil manejo, visando proporcionar uma redução de custos, uma vez que até então os equipamentos posturográficos disponíveis no mercado eram apenas por importação e apresentavam custos elevados. A plataforma em questão foi oficialmente lançada em 2017 e tem sido foco de estudos^(9-11,18)^, pois permite a realização da avaliação do equilíbrio corporal e de reabilitação por meio de jogos^(19)^. Entretanto, na população infantil, ainda não há estudos apresentando sua utilização e valores normativos^(7)^.

Utilizando a posturografia dinâmica com realidade virtual, Balance Rehabilitation Unit -BRU™, estudo analisou as respostas de crianças na faixa etária de 7 a 12 anos, separadas em grupos com e sem dificuldade escolar, referindo destaque para a limitação na realização da análise dos dados, devido a falta de padronização para crianças^(12)^. Todavia, foi possível observar diferença entre os grupos para as medidas de velocidade de oscilação e área de deslocamento do centro de pressão^(12)^. Pesquisadores acreditam que para a adequada análise dos resultados na posturografia, faz-se importante a padronização dos dados selecionados e considerados, visto que o estatocinesigrama e o estabilograma geram variáveis muitas vezes redundantes e desnecessárias ao examinador, assim, sugerem a utilização das principais variáveis referentes à área e à velocidade média total nas duas direções, anteroposterior e médio-lateral, em relação ao CP^(2)^.

Diferente estudo realizado com a plataforma de posturografia dinâmica Foam-Laser em crianças de 6 a 10 anos demonstrou que, independente do sexo, crianças apresentam respostas inferiores aos adultos, destacando o efeito da maturação no equilíbrio corporal^(20)^. Os achados da presente pesquisa, em equipamento do tipo estático, demonstraram mudanças nos valores médios com o passar da idade para os dados de EC e velocidades anteroposterior e média-lateral (Tabela 2) nas sete condições de exame, com redução dos valores conforme o aumento da idade, indicativo de evolução gradativa na maturação do equilíbrio corporal. O que vem ao encontro do estudo citado anteriormente^(20)^, que apesar de ter avaliado uma faixa etária diferente, demonstrou o efeito da maturação no controle postural por meio da comparação das respostas entre crianças e adultos. Dessa forma, reforça-se a necessidade da análise dos dados separadamente conforme a faixa etária, bem como a importância da padronização das respostas da posturografia em crianças de 4 a 6 anos de idade.

Segundo Ferreira et al.^(10)^ e Nishino et al.^(11)^ na análise das respostas em adultos, utilizando o mesmo equipamento de posturografia do presente estudo, Horus®, o limite de estabilidade deve ser considerado distinto por sexo e faixa etária, pois sujeitos do sexo masculino obtiveram respostas com valores superiores ao sexo feminino. Nossos achados corroboram em parte, pois observou-se diferença intra-grupo por sexo apenas para idade de 4 anos (Tabela 1), sendo os valores superiores nos meninos. Acredita-se que este achado possa ter relação com a diferença na altura dos sujeitos, pois os meninos apresentaram-se mais altos, em média, apenas nesta faixa etária.

Na análise das respostas de crianças de 3 a 6 anos de idade no exame de posturografia dinâmica Equitest®, pesquisadores encontraram diferença estatisticamente significativa na comparação das respostas entre as crianças de 3 e 4 anos, quando comparadas com as de 5 e 6 anos, verificando maior oscilação nas crianças menores, sendo assim, consideradas menos estáveis^(21)^. A presente pesquisa, realizada na faixa etária de 4 a 6 anos, corrobora com os pesquisadores, mesmo utilizando posturografia do tipo estática, pois os valores relacionados à oscilação corporal e ao deslocamento foram superiores nas crianças mais jovens. Além disso, as respostas observadas no EFR aumentaram o valor da porcentagem conforme o aumento da idade, nas sete condições de exame (Tabela 2). Outros pesquisadores afirmam que na posturografia, a estabilidade corporal é representada pelo dado de ERF, podendo ser observado quantitativamente e fornecendo a área ainda disponível para a oscilação do sujeito^(18)^. Quanto mais próximo a 100% maior será a estabilidade corporal na condição de exame avaliada^(18)^.

Segundo pesquisadores, a maturação dos sistemas sensoriais envolvidos com o equilíbrio corporal ocorre respeitando a seguinte ordem: sistema visual, seguido do sistema proprioceptivo e, por último, ocorre no sistema vestibular, chegando à maturidade funcional aos nove anos de idade^(22)^. Outra pesquisa concluiu que a transição para respostas semelhantes ao padrão do adulto não está completa na idade de 6 anos, para todas as condições sensoriais^(21)^. Nos resultados da presente pesquisa (Tabela 2), nas condições de avaliação onde havia demanda da dependência visual, houve diferença significativa entre as idades, com redução dos valores médios conforme o aumento da idade e, ocorreu o inverso para o ERF.

A mudança de respostas com o aumento da idade foi observada em pesquisa utilizando a plataforma *Wii Balance Board* e um *software* de análise criado para o próprio estudo, em 38 crianças e adolescentes saudáveis, com idades entre 3 e 14 anos^(13)^. A pesquisa afirmou uma tendência para uma diminuição significativa dos valores encontrados no exame de equilíbrio postural realizado em pé sob a plataforma com os olhos abertos até os 8 anos de idade, pois a variação do centro de pressão diminuiu significativamente com o aumento da idade da criança^(13)^. Estatisticamente, tal achado não foi frequente em todas as condições de exame na comparação entre as idades (Tabela 2), possivelmente por se tratar de plataforma de posturografia diferente da utilizada no presente estudo.

Os mesmos autores citados anteriormente relataram que, na verificação das respostas nas condições de teste com olhos abertos e com olhos fechados, independentemente da idade, os valores numéricos que descrevem a oscilação corporal aumentaram significativamente quando a criança estava de olhos fechados^(13)^. Na observação dos resultados comparando as condições de exame C1 com C2 e C3 com C4 (Tabela 2), percebe-se um aumento dos valores médios quando a criança estava em condições de exame sem o apoio visual. Apesar de a diferença ter sido sutil, ocorre assim o reforço da afirmação de que a falta do apoio visual, em crianças pequenas, piora o controle postural.

Na avaliação de crianças de 3 a 6 anos, foi observado que tanto para condição C1, quanto para condição C2, olhos abertos e olhos fechados, respectivamente, a estabilidade postural aumentou com a idade^(21)^. Quanto à análise por sexo e às respostas verificadas por sistemas, utilizando a plataforma de força *AMTI AccuSway Plus ACS*, verificou-se que o sistema visual é utilizado de forma diferente entre meninas e meninos em crianças e adolescentes^(23)^. Outro estudo traz que, além da questão maturacional, o controle da estabilidade corporal em meninas possui seu próprio ritmo de desenvolvimento, na faixa etária dos 11 aos 13 anos, demonstrando estar em fase de desenvolvimento individual na adolescência^(24)^. No presente estudo, houve diferença nas respostas entre meninos e meninas (Tabela 3), onde as meninas demonstraram valores menores para a velocidade de deslocamento em relação ao CP, sendo mais estáveis, bem como menor valor para o EC, correspondente à área de deslocamento do corpo sem oscilação. Esses resultados reforçam a importância da existência de padronização das respostas em avaliação do equilíbrio corporal em crianças, para adequado diagnóstico, tratamento e também para a utilização no monitoramento do desenvolvimento e/ou da efetividade terapêutica.

O controle do equilíbrio corporal sofre mudanças com o passar da idade, mudando de primariamente visual-vestibular para somatossensorial-vestibular, de acordo com estudo realizado utilizando uma posturografia dinâmica computadorizada^(21)^. Observou-se que, para a posturografia do tipo estática Horus®, nos achados da atual pesquisa, houve redução do valor de desvio padrão em cada condição de teste para as medidas sensoriais (Tabela 4), com o aumento da idade, demonstrando menor dispersão dos valores e, assim, efeito maturacional. Sabe-se que o desenvolvimento motor da criança é gradativo, conforme confirmado pelos resultados encontrados. A posturografia Horus® mostrou-se eficaz para determinar a evolução do equilíbrio em crianças pequenas, sem queixas vestibulares/auditivas, podendo, portanto, auxiliar no diagnóstico de crianças com alteração no equilíbrio.

Em crianças, percebe-se que até os 4 anos de idade elas conseguem reavaliar as informações multissensoriais e, após os 4 anos, ocorre uma reponderação intermodal, caracterizada como a fusão adaptativa de duas condições sensoriais, onde verificou-se que a resposta postural para uma condição foi dependente da amplitude da outra condição^(25)^. Estudos referem que a interdependência completa dos sistemas sensoriais ocorre em períodos diferentes, sendo considerado o sistema somatossensorial o que se desenvolve de forma mais precoce, estando semelhante ao adulto na idade entre 3 e 4 anos^(15,26)^, sendo mais tardia a maturação dos sistemas visual e vestibular^(15)^. Os achados atuais mostraram ausência de diferença estatística para os sistemas visual e somatossensorial (Tabela 4), na faixa estudada de 4 a 6 anos e 11 meses no equilíbrio corporal para a plataforma estática Horus®, demonstrando a presença, ainda, da dependência desses sistemas para o equilíbrio corporal nesta idade.

A dificuldade em se encontrar estudos que tenham utilizado a posturografia do tipo estática em crianças gerou a necessidade de comparação com a posturografia do tipo dinâmica durante a discussão. Considerou-se este fator como uma das limitações do estudo. Outrossim, ressalta-se a escassez de estudos para a plataforma Horus®, por se tratar de um equipamento novo no mercado. A partir dos valores propostos, sugere-se que novas pesquisas sejam realizadas com o intuito de investigar as respostas em crianças com desenvolvimento atípico e/ou queixas vestibulares.

## CONCLUSÃO

Foi possível estabelecer valores normativos para a posturografia estática Horus® em crianças hígidas de 4 a 6 anos de idade. Adicionalmente, sugere-se que, para esta população e ferramenta de avaliação, as respostas obtidas no exame sejam analisadas por faixa etária e sexo.

A posturografia computadorizada Horus® mostrou-se aplicável e eficaz para determinar a evolução do equilíbrio corporal em crianças pequenas, na faixa etária de 4 a 6 anos e 11 meses, com desenvolvimento típico e sem queixas vestibulares e/ou auditivas. Adicionalmente, acredita-se que esses achados podem auxiliar no diagnóstico de crianças com alterações no equilíbrio corporal.
